# Monoclonal Antibody Therapies for Hematological Malignancies: Not Just Lineage-Specific Targets

**DOI:** 10.3389/fimmu.2017.01936

**Published:** 2018-01-17

**Authors:** Carlos Cuesta-Mateos, Ana Alcaraz-Serna, Beatriz Somovilla-Crespo, Cecilia Muñoz-Calleja

**Affiliations:** ^1^Servicio de Inmunología, Instituto de Investigación Sanitaria Hospital Universitario de La Princesa, Madrid, Spain; ^2^IMMED S.L., Immunological and Medicinal Products, Madrid, Spain; ^3^Department of Immunology and Oncology, Centro Nacional de Biotecnología, Consejo Superior de Investigaciones Científicas (CSIC), Madrid, Spain

**Keywords:** monoclonal antibody, immunotherapy, hematological malignancies, non-lineage antigens, mechanism of action

## Abstract

Today, monoclonal antibodies (mAbs) are a widespread and necessary tool for biomedical science. In the hematological cancer field, since rituximab became the first mAb approved by the Food and Drug Administration for the treatment of B-cell malignancies, a number of effective mAbs targeting lineage-specific antigens (LSAs) have been successfully developed. Non-LSAs (NLSAs) are molecules that are not restricted to specific leukocyte subsets or tissues but play relevant pathogenic roles in blood cancers including the development, proliferation, survival, and refractoriness to therapy of tumor cells. In consequence, efforts to target NLSAs have resulted in a plethora of mAbs—marketed or in development—to achieve different goals like neutralizing oncogenic pathways, blocking tumor-related chemotactic pathways, mobilizing malignant cells from tumor microenvironment to peripheral blood, modulating immune-checkpoints, or delivering cytotoxic drugs into tumor cells. Here, we extensively review several novel mAbs directed against NLSAs undergoing clinical evaluation for treating hematological malignancies. The review focuses on the structure of these antibodies, proposed mechanisms of action, efficacy and safety profile in clinical studies, and their potential applications in the treatment of hematological malignancies.

## Introduction

Cancer treatment is expanding from non-specific cytotoxic chemotherapies to targeted therapies as a consequence of increased knowledge of the pathogenesis of cancer that leads to a better design of treatments to inhibit tumor growth and spread. Most of these therapies consist in monoclonal antibodies (mAbs) that bind to specific antigens (Ags) expressed on the surface of cancer and normal cells, mediating different mechanisms of action (MOA).

IgG antibodies, which are the most commonly used in cancer immunotherapy, show two regions that determine their biologic properties: the variable fragment (Fv), responsible for interaction with Ag and the constant fragment (Fc), responsible for interaction with immune cells or molecules bringing together cells bearing the Ag (or the Ag itself) to components of innate or acquired immunity. The Fc of an antibody is responsible for half-life, antibody-dependent cell-mediated cytotoxicity (ADCC), antibody-dependent phagocytosis (ADCP), or complement-dependent cytotoxicity (CDC) (1, 2). Both Fv and Fc determine the different and characteristics of MOA displayed by a single mAb and its utility as immunotherapeutic agent in cancer. These MOA may work alone or combined. Briefly, a particular mAb may inhibit ligand–receptor interactions, and/or induce proapoptotic signaling, and/or activate innate immune cells or molecules triggering ADCC, ADCP, or CDC, and/or may induce tumor cell killing by targeting regulatory molecules on host immune cells (1, 2). In addition, mAbs can be used to deliver payloads such as cytotoxic agents, toxins, or radioisotopes, which are coupled to the mAb targeting tumor cells (3). One explanation to the rapid growth of mAbs as therapeutic drugs is their plasticity. Antibodies can be engineered at several levels leading to customized modulations in the Fv/Fc properties. Altering the glycosylation status is the most extended modification among all the novel mAbs under development and is used to regulate anti- and proinflammatory properties and to control the binding to Fc receptors (FcRs) to modulate ADCC (4, 5).

In the hematological malignancies field, therapeutic mAbs are especially relevant owing to accessibility to tumor cells, facilitating *in vitro* studies of targets and MOA. In addition, the historical knowledge of the hematopoietic differentiation Ags, usually grouped as cluster of differentiation (CD) Ags, has provided a large number of potential targets in hematological malignancies. Similar to other cancers, tumor-associated Ags recognized by therapeutic mAbs in blood cancers fall into different categories. Many of them are present at the different normal maturation steps of a given linage and this is why they are called lineage-specific antigens (LSAs). For example, B-cell differentiation is associated with the expression of CD19, CD20, CD22, and surface Ig (6). Similarly, myeloid differentiation is associated with CD33 expression (7), whereas CD3 is the hallmark of the T-cell linage (8). These LSAs show significant overlapping expression patterns between leukemia or lymphoma subtypes within the same lineage.

It could be said that most of the LSAs are clinically validated targets in antibody-based therapy. CD20 is a LSA exclusively expressed on B-cells membrane and on the majority of malignant B-cells (6, 9). The “blockbuster” antibody rituximab is the first-in-class anti-CD20 mAb approved for the treatment of B-cell non-Hodgkin lymphoma (B-NHL) and chronic lymphocytic leukemia (CLL); it is by far the most important mAb used in hematological malignancies (10–12). Since its approval in 1997, four additional mAbs targeting different CD20 epitopes and displaying several MOA have been approved by the US Food and Drug Administration (US-FDA) (13–15). These CD20-targeting therapeutic mAbs account for >30% of all current therapeutic mAbs for cancer (3) and reflect the previous tendency to develop improved antibodies against the same LSAs. The MOA of antibodies directed to CD20 are given in Table 1.

**Table 1:** Characteristics of antibodies directed to CD20.

Target	mAb (commercial name/originator)	IgG class	MOA (compared to RTX)	Type—generation	Active indications in HMs (highest phase)	Reference
CD20	Rituximab (*Rituxan*/Biogen Idec)	Ch IgG1	CDC	Type I	Approved (B-NHL, CLL)	(11, 12)
			ADCC	First		
			PCD			

	Ofatumumab (*Arzerra*/Genmab)	Fh IgG1	↑ CDC	Type I	Approved (CLL)	(13, 16)
			~ ADCC	Second	I–II (HL)	
			↓ PCD		I–II–III (B-NHL)	

	Veltuzumab, IMMU-106 (Immunomedics)	Hz IgG1	↑ CDC	Type I	I–II (CLL, B-NHL)	(17, 18)
			~ ADCC	Second	Granted (ITP)	
			~ PCD			

	Ocrelizumab (*Ocrevus*/Biogen Idec; Genentech)	Hz IgG1	↓ CDC	Type I	I–II (FL) disc.	(19)
			↑ ADCC	Second	Approved (MS)	
			~ PCD			

	Obinutuzumab (*Gazyva/*Roche Glycart Biotech)	Hz IgG1	↓ CDC	Type II	Approved (CLL)	(20, 21)
		Glyco-Fc	↑ ADCC	Third	I–II–III (B-NHL)	
			↑PCD*			

	Ocaratuzumab, LY2469298 (Applied Molecular Evolution)	Hz IgG1	~ CDC	Type I	I–II (FL)	(22)
		Glyco-Fc	↑ ADCC	Third		
			~ PCD			

	Ublituximab, LFB-R603 (LFB Biotech.; rEVO Biologics)	Ch IgG1	~ CDC	Type I	III (CLL)	(23)
		Glyco-Fc	↑ ADCC	Third	I–II (B-NHL)	
			~ PCD			

*Antibodies that reached clinical studies. Biosimilars and immunoconjugates are excluded*.

RTX, rituximab; mAb, monoclonal antibody; MOA, mechanisms of action; HMs, hematological malignancies; Ch, human–mouse chimeric; Fh, fully human; Hz, humanized; Glyco-Fc, glycoengineered Fc fragment; ↑, higher; ↓, lower; ~ comparable; CDC, complement-dependent cytotoxicity; ADCC, antibody-dependent cell-mediated cytotoxicity; PCD, classical programmed cell death; PCD*, non-classical PCD; type I antibodies draw CD20 into lipid rafts, induce CDC, ADCC, and PCD; type II antibodies induce ADCC, PCD* but not CDC; B-NHL, B-cell non-Hodgkin’s lymphoma; HL, Hodgkin’s lymphoma; CLL, chronic lymphocytic leukemia; FL, follicular lymphoma; MS, multiple sclerosis; ITP, immune thrombocytopenic purpura; disc., discontinued in hematological malignancies.

The success of anti-CD20 mAbs has encouraged drug developers to propose novel LSAs, such as CD19, CD22, or CD79b (Table 2) (24–26). Despite these LSAs representing potential candidates for the treatment of B-cell cancers, antibodies directed to CD19 (MOR00208, inebilizumab, or MDX-1342) or CD22 (epratuzumab) have yielded only modest responses in clinical studies (9). This low efficacy has been attributed to high Ag internalization rates on mAb ligation (3). Consequently, CD19, CD22, and CD79 have been widely investigated for immunoconjugate therapy with promising clinical results as a single agent with no unexpected safety concerns. Finally, but beyond the scope of this review, it should be mentioned that other antibody formats, such as the bispecific T-cell engager (BiTe) blinatumomab, show promising results when targeting CD19 (27, 28). Thanks to a dual specificity for CD19 and CD3, this BiTe efficiently redirects host T-cells to CD19 expressed in tumor B-cells, although it shows neurological toxicity as treatment-related adverse event (29, 30).

**Table 2:** Characteristics of antibodies directed to LSAs.

Target	mAb (commercial name/originator)	IgG class	MOA	Active indications in HMs (highest phase)	Reference
CD19	Inebilizumab, MEDI 551 (Cellective Therapeutics)	Hz IgG1	ADCC	II (CLL/NCT01466153 ^a^C)	(31)
		Glyco-Fc	CDC	II (DLBCL/NCT01453205 ^a^C) disc.	
			ADCP	I–II (B-NHL/NCT02271945 ^a^C)	
				I (MM/NCT01861340 ^a^C)	

	MOR00208, XmAb5574 (Xencor)	Hz IgG1	ADCC	III (DLBCL/NCT02763319 ^a^R)	(24, 32)
			ADCP	II (BALL/NCT02763319 ^b^T)	
			PCD	II (CLL/NCT02639910 ^a^R)	
				II (B-NHL/NCT01685008 ^b^ANR)	

	MDX-1342 (Medarex)	Fh IgG1	ADCC	I (CLL/NCT00593944 ^b^C)	(33)
		Glyco-Fc			

CD22	Epratuzumab, AMG-412, IMMU-103 (Immunomedics)	Hz IgG1	ADCC	II (B-ALL/NCT01802814 ^b^R)	(25)
			PCD	III (B-NHL/NCT00022685 ^b^C)	
			Alterations in CD22 and BCR signaling its action	II (FL/NCT00553501 ^a^C)	

*Antibodies that reached clinical studies. Biosimilars and immunoconjugates are excluded*.

*^a^Combined therapy*.

*^b^Monotherapy*.

*mAb, monoclonal antibody; MOA, mechanisms of action; HMs, hematological malignancies; Ch, human–mouse chimeric; Fh, hully human; Hz, humanized; Glyco-Fc, glycoengineered Fc fragment; CDC, complement-dependent cytotoxicity; ADCC, antibody-dependent cell-mediated cytotoxicity; ADCP, antibody-dependent cell-mediated phagocytosis; PCD, programmed cell death; B-NHL, B-cell non-Hodgkin’s lymphoma; CLL, chronic lymphocytic leukemia; DLBCL, diffuse large B-cell lymphoma; MM, multiple myeloma; FL, follicular lymphoma; B-ALL, B-cell acute lymphoblastic leukemia; BCR, B-cell receptor; DISC., discontinued; NCT, number of clinical trial (clinicaltrials.gov); C, completed; R, recruiting; T, terminated; ANR, active non-recruiting*.

A different group of tumor Ags are the non-lineage-specific antigens (NLSAs), which comprise many molecules that are preferentially expressed by tumor cells but not restricted to specific leukocyte subsets or tissues and include, among others, oncogenic receptors, chemokine receptors (CKRs), and molecules involved in the formation and preservation of the tumor microenvironment (TME). The ubiquous expression of many NSLAs potentially enables antibodies directed to these molecules to be used in different hematological malignancies, or even universally in cancer.

Limited clinical efficacy of some mAbs targeting LSAs and the advent of patients with refractory diseases to therapies directed to LSAs boosted the research on many NLSAs with a relevant role in the pathogenesis of cancer, especially in B-cell malignancies (9, 34). Moreover, in some disorders the lack or loss of LSA expression in cell membrane may preclude the use of antibodies, thus prompting research of other potential therapeutic targets. This is the case of multiple myeloma (MM), a B-cell disorder where tumor cells do not express CD20 (35) and where novel antibodies directly targeting several NLSAs are a profound change compared with earlier treatment approaches based on anti-CD20 antibodies.

Efforts to target NLSAs have resulted in an ever-increasing number of new murine, chimeric and human antibodies with proven efficacy in preclinical models. Here, we extensively review the results of several novel mAbs directed against NLSAs undergoing clinical evaluation (Table 3). The review focuses on the structure of these antibodies, proposed MOA, efficacy, and safety profile in clinical studies, and their potential applications in the treatment of hematological cancers.

**Table 3:** Characteristics of antibodies directed to NLSAs.

	Target	mAb (commercial name/originator)	IgG class	MOA	Active indications in HMs (highest phase)
Glycoproteins and oncogenic recepteors	CD52	Alemtuzumab (*Campath*/University of Cambridge)	Hz IgG1	ADCC	Approved (CLL)
				CDC	II (T-PLL/NCT01186640 ^a^C)
				ADCP?	
					II (PTCL/NCT01806337 ^a^C)

	CD38	Daratumumab, JNJ-54767414 (*Darzalex*/Genmab)	Hz IgG1	ADCC	Approved (MM)
				CDC	II (MCL, DLBCL, FL/NCT02413489 ^a^T)
				ADCP	
				Blocks CD38	

		Isatuximab, SAR650984 (ImmunoGen)	Hz IgG1	ADCC	III (MM/NCT02990338 ^b^R)
				CDC	II (T-ALL, T-NHL/NCT02999633 ^b^R)
				ADCP	
				Blocks CD38	

		MOR202, MOR03087 (MorphoSys)	Hz IgG1	ADCC	I–II (MM/NCT01421186 ^b^R)
				ADCP	
				Blocks CD38	

	SLAMF7 (CS1, CD319)	Elotuzumab, HuLuc63, BMS-901608 (*Empliciti*/PDL BioPharma)	Hz IgG1	ADCC	Approved (MM)

	CD37	BI836826 (Boehringer Ingelheim)	Ch IgG1	ADCC	II (DLBCL/NCT02624492 ^b^R)
			Glyco-Fc	PCD	I (CLL/NCT01296932 ^a^C, NCT02538614 ^b^T)

		Otlertuzumab, TRU-016 (Trubion Pharmaceuticals)	Fv-Fc	ADCC	I–II (CLL/NCT01188681 ^b^C)
				PCD	I (B-NHL/NCT00614042 ^a^C)

	CD98 (4F2, FRP-1)	IGN523 (Igenica)	Hz IgG1	ADCC	I (AML/NCT02040506 ^a^C)
				CDC	
				PCD	

	DKK-1	BHQ880 (MorphoSys; Novartis)	Fh IgG1	Blocks DKK-1	II (MM/NCT01302886 ^a^C, NCT01337752 ^a^C)
			GlycoFc		
				ADCC	

		DKN-01, LY-2812176 (Eli Lilly)	Hz IgG4	Blocks DKK-1	I (MM/NCT01711671 ^b^C, NCT01457417 ^b^C)

	CD157 (BST-1)	OBT357, MEN1112 (Menarini; Oxford BioTherapeutics)	Hz IgG1	ADCC	I (AML/NCT02353143 ^a^R)
			GlycoFc		

	GRP78 (BiP)	PAT-SM6 (OncoMab GmbH)	Fh IgM	CDC	I (MM/NCT01727778 ^a^C)
				PCD	

	TRAIL-R1 (DR4)	Mapatumumab, TRM1, HGS-1012 (Cambridge Antibody Technology)	Fh IgG1	PCD	I (NHL, HL/NCT00094848 ^a^C) disc.
					II (MM/NCT00315757 ^b^C) disc.

	ROR-1	Cirmtuzumab, UC-961 (University of California, San Diego)	Hz IgG1	PCD	I–II (CLL, MCL/NCT03088878 ^b^NYR)
				Blocks ROR-1	
					I (CLL/NCT02860676 ^a^E, NCT02222688 ^a^R)

	Notch-1	Brontictuzumab, OMP-52M51 (OncoMed Pharmaceuticals)	Hz IgG2	Blocks Notch-1	I (HM/NCT01703572 ^a^C) disc.

	TfR1 (CD71)	E2.3/A27.15 (University of Arizona)	mIgG1	Blocks TfR1	I (HM/NCT00003082 ^a^C)

	EPHA3	Ifabotuzumab, KB004 (Ludwig Institute for Cancer Research)	Hz IgG1	ADCC	I–II (AML/NCT01211691 ^a^S)
				PCD	

	HLA-DR	IMMU114, hL243 (Immunomedics)	Hz IgG4	PCD	I (B-NHL/NCT01728207 ^a^R)

	G(M2)	BIW-8962 (Kyowa Hakko Kirin Co.)	Hz IgG1	ADCC	I (MM/NCT00775502 ^a^T)

Chemokine receptors	CCR4	Mogamulizumab, KW-0761 (*Poteligeo*/Kyowa Hakko Kirin Co.)	Hz IgG1	ADCC	Approved (ATL, CTCL, PTCL)
					II (NK-lymphoma/NCT01192984 ^a^C)

	CXCR4	Ulocuplumab, BMS-936564, MDX-1338 (Medarex)	Hz IgG4	Blocks CXCR4	I–II (AML/NCT02305563 ^b^R)
					I (MM/NCT01359657 ^b^C)
					I (CLL, DLBCL, FL/NCT01120457 ^a^C)

		PF-06747143 (Pfizer)	Hz IgG1	ADCC	I (AML/NCT02954653 ^b^R)
				CDC	
				Blocks CXCR4	

Soluble factors and associated receptors	BAFF	Tabalumab, LY2127399 (Eli Lilly)	Fh IgG4	Blocks BAFF	II (MM/NCT01602224 ^b^C) disc.

	BAFF-R	VAY736 (MorphoSys; Novartis)	Fh IgG1	Blocks BAFF-R	I (CLL/NCT02137889 ^a^T)
				ADCC	
			GlycoFc		

	RANKL	Denosumab, AMG-162 (*Prolia; Ranmark; Xgeva*/Amgen)	Fh IgG2	Blocks RANKL	III (MM/NCT01345019 ^a^ANR)
					II (MM/NCT00259740 ^a^C, NCT02833610 ^a^R)
					II (NHL with hypercalcemia/NCT00896454 ^a^C)

	IL-6	Siltuximab, CNTO-328 (*Sylvant*/Centocor)	Ch IgG1	Blocks IL-6	II (MM/NCT00911859 ^b^C, NCT00402181 ^a^C)

	IL-6R	Tocilizumab, R-1569 (*Actemra*/Chugai Pharmaceutical; Osaka University)	Hz IgG1	Blocks IL-6R	I (CLL/NCT02336048 ^b^R)
					I (MM/NCT02447055 ^b^W) disc.

	IL-3Rα (CD123)	CSL360 (CSL)	Ch IgG1	ADCC	I (AML/NCT00401739 ^a^C)
				CDC	
				Blocks IL-3Rα	

		Talacotuzumab, JNJ-56022473, CSL362 (CSL)	Hz IgG1 GlycoFc	ADCC	III (AML/NCT02472145 ^b^ANR)
				Blocks IL-3Rα	
					II (MDS/NCT03011034 ^b^R)

		XmAb14045 (Xencor)	Fh IgG1	ADCC	I (AML, B-ALL, DC Neoplasm, CML/NCT02730312 ^a^R)

		KHK2823 (Kyowa Hakko Kirin Co.)	Fh IgG1	ADCC	I (AML, MDS/NCT02181699 ^a^ANR)

	IL-2Rα (CD25)	Basiliximab, SDZ-CHI-621 (*Simulect*/Novartis)	Ch IgG1	Blocks IL-2Rα	II (AML, CML, ALL, CLL, HL, MM/NCT00975975 ^a^C)
		Daclizumab	Hz IgG1	Blocks IL-2Rα	II (ATL/NCT00001941 ^a^C)
					II (MM, NHL/NCT00006350 ^b^C)

	IGF-1R (CD221)	Ganitumab, AMG-479 (Amgen)	Fh IgG1	Blocks IGF-1R	I (NHL/NCT00562380 ^a^C)
		Figitumumab, CP-751871 (Pfizer)	Fh IgG2	Blocks IGF-1R	I (MM/NCT01536145 ^a^C) disc.
		Dalotuzumab, MK-0646 (Pierre Fabre)	Hz IgG1	Blocks IGF-1R	I (MM/NCT00701103 ^a^C) disc.
		AVE1642 (ImmunoGen)	Hz IgG1	Blocks IGF-1R	I (MM/NCT01233895 ^a^C) disc.

	GM-CSF (CSF2)	Lenzilumab, KB003 (KaloBios Pharmaceuticals)	Hz IgG1	Blocks GM-CSF	I–II (CMML/NCT02546284 ^a^R)

	HGF	Ficlatuzumab, AV-299 (AVEO Pharmaceuticals)	Hz IgG1	Blocks HGF	I (NHL, HL, MM/NCT00725634 ^b^C)
					I (AML/NCT02109627 ^b^R)

Adhesion molecules	CD44	RG7356, RO5429083 (Chugai Biopharmaceuticals; Roche)	Hz IgG1	Blocks CD44	I (AML/NCT01641250 ^b^C)

	VLA-4 (CD49d)	Natalizumab, BG-0002-E (*Tysabri*/Elan Corporation)	Hz IgG4	Blocks VLA-4	I–II (MM/NCT00675428 ^a^T) disc.

	ICAM-1 (CD54)	BI-505 (BioInvent International)	Fh IgG1	ADCC	I (MM/NCT01025206 ^a^C)
				ADCP	II (sMM/NCT01838369 ^a^C)
				PCD	

Angiogenesis	VEGF-A	Bevacizumab (*Avastin*/Genentech; Hackensack University Medical Center)	Hz IgG1	Blocks VEGF-A	II (MM/NCT00482495 ^a^C, NCT00473590 ^b^C)
					II (CLL/NCT00290810 ^a^C, NCT00448019 ^b^C) disc.
					III (DLBCL/NCT00486759 ^b^T) disc.
					II (FL/NCT00193492 ^b^C)

	Endosialin (CD248, TEM1)	Ontecizumab, MORAB-004 (Ludwig Institute for Cancer Research; Morphotek)	Hz IgG1	Blocks endosialin	I (HM/NCT01748721 ^a^C)

*Antibodies that reached clinical studies. Biosimilars and immunoconjugates are excluded*.

*^a^Monotherapy*.

*^b^Combined therapy*.

*mAb, monoclonal antibody; MOA, mechanisms of action; HMs, hematological malignancies; Ch, human–mouse chimeric; Fh, fully human; Hz, humanized; m, mouse; Glyco-Fc, glycoengineered Fc fragment; CDC, complement-dependent cytotoxicity; ADCC, antibody-dependent cell-mediated cytotoxicity; ADCP, antibody-dependent cell-mediated phagocytosis; PCD, programmed cell death; B-NHL, B-cell non-Hodgkin’s lymphoma; HL, Hodgkin’s lymphoma; CLL, chronic lymphocytic leukemia; FL, follicular lymphoma; DLBCL, diffuse large B-cell lymphoma; MCL, mantle cell lymphoma; MM, multiple myeloma; B-ALL, B-cell acute lymphoblastic leukemia; T-PLL, T-cell prolymphocytic leukemia; PTCL, peripheral T-cell lymphoma; CTCL, cutaneous T-cell lymphoma; ATL, adult T-cell leukemia and lymphoma; AML, acute myeloid leukemia; CML, chronic myeloid leukemia; CMML, chronic myelomonocytic leukemia; MDS, myelodysplastic syndrome; DC, dendritic cells; disc., discontinued in hematological malignancies; NCT, number of clinical trial (clinicaltrials.gov); C, completed; R, recruiting; T, terminated; ANR, active non-recruiting; NYR, not yet recruiting; E, enrolling by invitation; T, terminated; S, suspended; W, withdrawn*.

## Antibodies Targeting Glycoproteins and Oncogenic Receptors

Pathologic clonal populations express or overexpress different NLSAs which are involved in different oncogenic pathways and may confer an evolutionary advantage to the tumor. In some cases, high expression of the NLSAs is the rationale behind targeting a single receptor. In other cases, this targeting represents an optimal strategy to avoid cancer cell proliferation and survival.

### CAMPATH-1 (CD52)

CD52 is a glycoprotein anchored to glycosylphosphatidylinoitol (GPI) present on the surface of mature lymphocytes, monocytes and dendritic cells (DCs) (36). CD52 expression is particularly high on T-cell prolymphocytic leukemia (T-PLL), Sézary syndrome (SS), acute lymphoblastic leukemia (ALL), CLL, and acute myeloid leukemia (AML) (36–39), which is the reason why it was selected as therapeutic target despite not having a clear role in the pathogenesis of these conditions. Nonetheless, efficacy as single agent in patients with high-risk CLL (40–42) prompted the approval of the anti-CD52 mAb alemtuzumab as front-line therapy in CLL. The main MOA of alemtuzumab are CDC and ADCC (36) which are likely to be involved in its efficacy in SS and T-PLL (39). Curiously, side effects associated with immune-suppression and infections were more frequent in B-cell than in T-cell malignancies, probably due to off-target activities (43). Despite being one of the few working weapons in T-cell malignancies, alemtuzumab was withdrawn in 2012, due to a strategic decision, and now is only available through an international compassionate use program.

### CD38

In some leukocytes, this type II transmembrane glycoprotein behaves both as an ectoenzyme (NADase/ADPR cyclase) and as a receptor involved in cell adhesion, calcium flux and signal transduction (44, 45). While its expression was low to moderate on lymphoid and myeloid cells, both normal and tumor plasma cells exhibited high levels of CD38, making it an attractive target for MM (44, 45). In 2015, daratumumab, a humanized anti-CD38 IgG1 mAb, became the first mAb approved for MM (46). In preclinical models, daratumumab caused cell death through ADCC, CDC, ADCP, and blocking of CD38 that inhibits its enzymatic activity and induces apoptosis in a caspase-dependent manner (47–50). In addition, it has been recently suggested that depletion of CD38^+^ immunosuppressive regulatory T (Tregs) and B-cells and myeloid-derived suppressor cells (MDSCs) increase antitumor effector T-cell responses (51). Altogether, these MOA are responsible for daratumumab single-agent efficacy as demonstrated by two phase I–II trials in pretreated MM patients (NCT00574288; NCT01985126) that prompted FDA approval of daratumumab (52, 53). Moreover, daratumumab shows promising results both in the relapsing/refractory setting (rrMM) and in the upfront setting when combined with other potent MM therapeutics, including lenalidomide, dexamethasone and bortezomib (54–56). As a result, the FDA granted “Breakthrough Therapy” designation to daratumumab in combination with lenalidomide–dexamethasone or bortezomib–dexamethasone for the treatment of previously treated MM.

In the light of the aforementioned results, it is not difficult to find several anti-CD38 mAbs under clinical development. Isatuximab, with similar MOA to daratumumab, has shown promising results in ongoing phase I–II studies in rrMM both in monotherapy (NCT01084252) (57) or combined with immunomodulatory drugs (IMIDs) or dexamethasone (NCT01749969) (58). Another mAb is MOR202, which lacks CDC activity, but still shows promising results in ongoing trials both in monotherapy or in combination (NCT04121186) (59, 60). Last, but not least, anti-CD38 mAbs are attracting the interest in many other B-cell malignancies expressing surface CD38 including CLL, mantle cell lymphoma (MCL), diffuse large B-cell lymphoma (DLBCL), and transformed follicular lymphoma (FL) (NCT02413489) (44, 52, 61).

### Signaling Lymphocytic Activation Molecule Family Member F7 (SLAMF7; CS1)

This glycoprotein is moderately expressed by normal plasma cells and by cytolytic lymphocyte subsets such as natural killer (NK) cells, NKT cells or CD8^+^ T-cells (62, 63). As their normal counterpart, MM plasma cells express SLAMF7, but at higher levels (62, 63) as a consequence of an amplification of chromosome 1q23 region, where SLAMF7 gene is located, which is very frequent in aggressive MM (62, 64). SLAMF7 expression in MM does not correlate with other high-risk cytogenetic abnormalities or the degree of disease progression (62, 63), thus validating SLAMF7 as a potential target.

The humanized IgG1 mAb elotuzumab was the first-in-class anti-SLAMF7 to be approved by the FDA in 2015, and the second antibody marketed for the treatment of MM (65). Similar to daratumumab, elotuzumab has several MOA *in vitro*, although it seems to predominantly act through ADCC *in vivo* (63, 66–68) since homozygosis for the high-affinity FcγRIIIa Val significantly prolonged median period free survival in clinical settings (69). In addition, elotuzumab is an agonistic mAb, which activates NK cells, further enhancing their cytotoxicity through a unique SLAM-associated pathway. Conversely, MM cells lack the SLAM-associated adaptor EAT-2 thus preventing proliferation upon elotuzumab binding (70, 71).

In contrast to daratuzumab, elotuzumab has demostrated limited activity as a single agent in both preclinical and clinical studies (63). The deffects on NK cell activity observed in MM patients may be explained by elotuzumab activity relying on ADCC. Also, the paradox of NK cells becoming targets may also contribute to the lack of objective responses in rrMM patients treated with elotuzumab as single-agent (72). Therefore, to reach its maximum efficacy, elotuzumab needs to be combined with other antimyeloma agents such as lenalidomide-dexamethasone (NCT00742560, NCT01239797) (66, 73, 74) or bortezomib-dexamethasone (69, 75). Currently, several studies are examining different combinations either in the upfront or the relapsed/refractory settings.

### CD37

This heavily glycosylated tetraspanin is highly expressed by mature B-cells and B-cell malignancies, including CLL and NHL (76–78). The exact function of CD37 has not yet been elucidated, although it seems to be important for T-cell-dependent B-cell responses, and may be involved in both pro- and antiapoptotic signaling (78). In addition, recent evidence confirms CD37 expression on the surface of CD34^+^/CD38^−^ AML stem cells (LSCs), which are considered the root of tumor drug resistance and recurrence (79). For this reason, despite initially conceived as a lineage-specific therapy for B-cell malignancies, anti-CD37 mAbs are also being tested as therapeutics in AML.

CD37 has unique properties for generating therapies as low internalization rates allows the preservation of its ADCC activity (76). For this reason, different kinds of IgG formats targeting CD37 are currently in clinical development. BI836826 is an Fc-engineered, chimeric IgG1 that mediates potent ADCC and induces apoptosis on CD37-overexpressing cells (80). This mAb is undergoing phase I–II studies for the treatment of CLL and B-NHL, either as a single agent or in combination with ibrutinib, idelalisib or rituximab. A number of anti-CD37 immunoconjugates are also in advanced clinical phases (79, 81, 82) (Table 6).

Of special interest is the modular homodimer called otlertuzumab (TRU-016) formed by a single-chain Fv linked to the hinge region and Fc domain of hIgG1 (148, 149). Otlertuzumab induces apoptosis directly *via* binding to the CD37 protein, which results in up-regulation of the proapoptotic protein BIM (also termed BCL2L11) (150). In addition, otlertuzumab triggers Fc-dependent cytotoxicity (ADCC) but does not induce complement activation. In B-cell malignancies, otlertuzumab has shown efficacy as a single agent or combined with other therapeutic drugs in preclinical models (151, 152) as well as in phase I (NCT00614042) and phase II (NCT01188681) studies (149, 153). Other studies in B-NHL patients (NCT01317901) further confirm that combination regimens are well tolerated and lead to higher response rates (154). As a consequence, novel clinical trials are recruiting patients to evaluate combinations with standards of care in B-NHL such as rituximab, obinutuzumab, idelalisib, and ibrutinib.

### CD98

The CD98 heterodimer consists of a type II single-pass transmembrane glycoprotein (also known as 4F2 Ag heavy chain or FRP-1) with two biochemical functions depending on the coupled light chain (155). Upon binding to the cytoplasmic tail of the integrin beta-chain it mediates adhesive signals thereby controlling cell proliferation, survival, migration, epithelial adhesion and polarity. In addition, CD98 contributes to the amino acid transport processes through the binding to one of the six permease-type amino acid transporters including L-type amino acid transporter 1 and 2 (LAT-1 and LAT-2) (155, 156), whose localization and proper function rely on the CD98 heavy chain (157). Both CD98-mediated activities take place on fast-cycling cells undergoing clonal expansion, such as AML cells, where CD98 supports elevated growth rates and contributes to proliferation, survival, and metastasis (158). Few approaches target metabolic cancer, and most of them are based on small molecules against CD98-associated light chains (158). In this context, targeting CD98 heavy chain with antibodies provides an alternative approach as demonstrated by IGN523, a novel humanized anti-CD98 mAb with robust preclinical activity against established lymphoma tumor xenografts (158). IGN523 elicits strong ADCC, mild CDC, and induces lysosomal membrane permeabilization that elicits capase-3- and caspase-7-mediated apoptosis in the presence of crosslinking antibody. But the most differential feature of IGN523 is the inhibition of essential amino acid (phenylalanine) uptake by rapidly proliferating tumor cells that ultimately results in caspase-3- and -7-mediated apoptosis (158). IGN523 has been evaluated in a completed Phase I study for rrAML (NCT02040506), although results are not published yet (158, 159).

### Dickkopf-1 Protein (DKK1)

This NLSA is related to the canonical Wnt/beta-catenin signaling pathway. DKK1 is a soluble inhibitor that binds simultaneously the transmembrane receptors Kremen-1 or 2 and the Wnt/beta catenin coreceptor LRP5/6 (160). This extracellular binding leads to endocytosis of the DKK1-associated complex that impairs a subsequent activation of Wnt/beta-catenin signaling. The first association between DKK-1 and cancer was described in MM patients suffering osteolytic lesions MM (160). Later, on preclinical studies have demonstrated that a neutralizing DKK mAb reduces osteolytic bone resorption, increases bone formation, and controls MM growth (161–163). BHQ880 and DKN-1 are neutralizing humanized IgG1 mAbs, which are being tested in phase I–II studies in MM. Most of the studies (NCT00741377, NCT01457417) are using anti-DKK-1 mAbs in combination with antimyeloma therapy, except the phase II study that evaluated the efficacy of BHQ880 in monotherapy in previously untreated patients with high risk smoldering MM (phase II, NCT01302886). Overall, BHQ880 was well tolerated but the clinical benefits were limited (164). For this reason, other studies were designed to test the efficacy of anti-DKK-1 antibodies in the setting of MM with bone alterations combined with specific agents such zoledronic acid (NCT00741377).

### CD157 (BST-1)

This GPI-linked membrane protein has a close resemblance to CD38 and a significant role in myeloid cells trafficking and pre-B-cell growth (165–167). It is, therefore, not surprising that high levels of CD157 can be found in B-ALL cells and in most primary AML patient samples, including the LSCs compartment (168). Based on this rationale, a novel defucosylated IgG1 termed OBT357/MEN1112 validated CD157 as a therapeutic target in AML *in vitro* and *ex vivo* models (168). Now, the potent ADCC observed in preclinical phases is under evaluation in a phase I study in AML patients (NCT02353143).

### Glucose-Regulated Protein 78 (GRP78; BiP; HSP5a)

Members of the heat shock protein-70 family, if expressed on the cell membrane, are NLSAs of interest in mAb-based cancer therapy. GRP78 is a protein with multiple functions related to its different cellular locations. It may control the unfolded protein response, the macroautophagia or prosurvival pathways activated by PI3K/AKT. In some circumstances like glucose starvation, hypoxia or protein malfolding, GRP78 is translocated to the membrane, where it mediates, in general, cytoprotective responses (169). Many tumor cells, including MM, overexpress GRP78 on the outer plasma membrane to promote tumor survival, proliferation, and motility and this overexpression correlates with an adverse prognosis and drug resistance (170). Interestingly, normal plasma cells do not express the molecule on their membrane (171). Based on this, GRP78 is an ideal candidate for immunotherapeutic intervention of MM. Recently, the natural fully human IgM PAT-SM6 (initially isolated from a patient with gastric cancer) was evaluated as monotherapy in a phase I study in rrMM (NCT01727778). Results show that PAT-SM6 is well tolerated but has modest clinical activity (169). PAT-SM6 lacks ADCC activity thus its MOA mainly relies on apoptosis and to a lesser extent CDC (171, 172). Interestingly, patients who received prior treatment with proteasome inhibitors responded much better to PAT-SM6 than patients who had been previously treated with IMIDs or other chemotherapeutics. Hence, future clinical studies will focus on synergistic combinations with proteasome inhibitors to induce better clinical responses (press release by Patrys).

### Tumor Necrosis Factor-Related Apoptosis-Inducing Ligand Receptor-1 (TRAIL-R1; DR4)

This protein, also known as death receptor 4, is a cell surface receptor that binds TRAIL (ApoL2) and activates the extrinsic apoptotic pathway (173). After binding of either ligand or agonist antibodies to TRAIL-R1, a death-inducing signaling complex (DISC) starts the recruitment, cleavage and activation of caspases-3, -6, -7, resulting in the characteristic programmmed cell death (PCD) (173). The expression of TRAIL-R1 is minimal or absent in healthy tissues. In contrast, this receptor is frequently detected in cancer, including B-cell malignancies (174–176). This rationale boosted the development of the agonist anti-TRAIL-R1 IgG1 antibody mapatumumab (HGS-ETR1). Unlike native TRAIL, mapatumumab has longer half-life and binds specifically to TRAIL-R1 and not to the other TRAIL receptors (177). Like TRAIL, mapatumumab mediates caspase-dependent apoptosis by binding TRAIL-R1. In preclinical models of hematological malignancies, mapatumumab induced apoptosis in a wide spectrum of human cancers and promoted tumor regressions in xenograft models (175–179). Interestingly, in a recent study, the combination of mapatumumab with low dose bortezomib potentiated the uptake of myeloma cell apoptotic bodies by DC and induced antimyeloma cytotoxicity by both CD8^+^ T-cells and NK cells (180). Based on this, it has been suggested that mapatumumab may also promote endogenous antitumor immune responses.

Results from a phase II study (NCT00094848) demonstrated that mapatumumab was capable of producing clinical responses when used as single agent in patients with B-NHL (181), particularly FL. Of interest, immunohistochemistry analysis suggested that strong TRAIL-R1 staining in tumor specimens was not a requirement for mapatumumab activity in FL (181). In another phase II study in MM (NCT00315757), no differences in efficacy were observed between patients receiving mapatumumab plus bortezomib and the control group. What remains unclear is whether immunosuppressive effects of bortezomib could affect the ability of mapatuzumab to promote immune responses (180).

### Receptor Tyrosine Kinase-Like Orphan Receptor 1 (ROR-1)

This type I membrane glycoprotein lacks catalytic activity but is essential for ligand binding and signal transduction in the non-canonical Wnt pathway. It is considered an oncofetal Ag since it is highly expressed during early embryonic development, where it modulates neurite growth, but absent in most adult tissues (182). Ubiquitously found in human cancers, ROR-1 protein is highly expressed on the surface of CLL cells and several other B-cell malignancies where it favors invasion, metastasis and therapeutic resistance (183).

Cirmtuzumab (UC-961) is the first IgG1 directed against a functional epitope of the extracellular domain of ROR-1. It blocks ROR-dependent non canonical Wnt5a signaling through ROR-1 dephosphorylation, thus blocking tumor cell proliferation, migration and survival, leading to tumor cell death by apoptosis (184, 185). Preclinical and phase I studies have shown good tolerability and moderate activity of cirmtuzumab when used as a single agent but with apparent synergistic activity with other agents like ibrutinib (184, 185). Interestingly, cirmtuzumab acts like other kinase inhibitors mobilizing ROR1-expressing CLL cells, thereby preventing progression in protective niches and providing an additional MOA (185). Currently, cirmtuzumab is facing phase I–II studies as single agent in rrCLL (NCT02222688) or in combination with ibrutinib in rrCLL or rrMCL (NCT03088878). Finally, the interest in ROR-1 as target is supported by two bi-specific antibodies (ROR1-CD3-DART and APVO425) that aim to redirect cytotoxic T-cells to ROR-expressing cells (182) and by a novel anti-ROR1 single-chain (sc) antibody able to induce apoptotic death of CLL lines and primary CLL cells (186).

### Notch-1

Aberrant signaling of this Notch family transmembrane receptor has been implicated in cancer, cancer stem cells, and tumor vasculature (187). Indeed, Notch1 is a well-characterized oncoprotein in T-ALL and lymphomas where activating Notch1 mutations are responsible for approximately 60% of T-ALL cases (188, 189). In preclinical models, blocking the extracellular region of Notch1 with antibodies decreased T-ALL tumor growth by inhibiting cancer cell growth and by disrupting angiogenesis (190, 191). Brontictuzumab (OMP-52M51) is the only anti-Notch-1 antibody tested in clinical settings (NCT01703572). Although completed, the results from this study have not been published and development in hematological cancers was discontinued. Severe grade adverse events could explain this, as Notch-1 inhibition causes gastrointestinal side effects (190, 191). Finally, antibodies against DLL4, a ligand of Notch-1, are alternatives to target this pathway, including OMP-21M18 or MEDI0639. Already tested *in vitro* and *in vivo* in solid tumors, they are currently under evaluation in several ongoing clinical trials. Nonetheless, no development has been reported for blood cancers (192).

### EphrinA3 (EPHA3)

This member of the ephrin subfamily of receptor protein-tyrosine kinases can be considered an oncofetal Ag since it is not expressed in normal healthy adult tissues but is overexpressed by a variety of tumor types instead, including most hematological malignancies (193), where it plays an important role in tumor cell proliferation. Ifabotuzumab (KB004) is a humanized, non-fucosylated IgG1 mAb targeting EphrinA3 which induces apoptosis and stimulates ADCC (193). One phase I–II study (NCT01211691) tested for the utility of KB004 as a single agent in patients with heavily pretreated AML. KB004 was well tolerated but the efficacy was very limited with responses observed in patients with fibrotic myeloid diseases (194). In this study, it was postulated that low expression of EPHA3 in various myeloid leukemic cell subsets or the ability of KB004 to be internalized upon Ag binding are likely explanations for KB004 ineffectiveness (194). Based on this ability to be internalized, an alternative approach targeting EPHA3 with an immunoconjugate was proposed (195).

### HLA-DR

Ligation of HLA-DR by antibodies is one of the oldest approaches to eliminate hematological tumors, since most of them express high levels of this MHC class II molecule (196). Anti-HLA-DR antibodies with different MOA such as apolizumab, Lym-1, and 1D09C3 eventually had no convincing clinical response in several clinical trials and were discontinued (197). In addition, anti–HLA-DR mAbs are potent inducers of complement activation, which plays a pivotal role in the pathogenesis of mAb infusion side effects (197). To our knowledge, there is only one ongoing phase I study recruiting patients to test the safety and efficacy of an anti-HLA-DR antibody called IMMU-114 in B-cell disorders (NCT01728207). This drug is a humanized IgG4 form of murine anti–human HLA-DR mAb, L243, which recognizes a conformational epitope in the alpha chain of HLA-DR. Due to safety concerns related to the expression of HLA-DR in non-tumor cells, IMMU-114 was specifically generated to kill tumor cells avoiding CDC or ADCC (198). IMMU-114 binding to tumor B-cells results in antiproliferative effects and apoptosis and has demonstrated efficacy in preclinical models (198). Although the exact mechanism has yet to be fully elucidated, it appears to induce hyperactivation of ERK- and JNK-dependent mitogen activated protein kinase signaling pathways that may lead to mitochondrial membrane depolarization and reactive oxygen species generation. This eventually leads to an induction of tumor cell apoptosis and a reduction in tumor cell proliferation (198).

## Antibodies Targeting Chemokine Receptors

Chemokines are small chemotactic cytokines that bind to specific surface seven transmembrane domain G protein-coupled receptors, or CKRs. Upon binding of their ligands, CKRs promote cell survival, proliferation, and adhesion, contributing to mammalian development and organogenesis, thus playing a central role in homeostasis and the maintenance of innate and acquired immunity (199). In cancer, CKRs may associate with tumor cells facilitating their survival, proliferation, and metastasis (200, 201). Moreover, they may also promote an immunotolerant milieu by recruiting Treg, tumor-associated macrophages (TAMs) or MDSCs that opens the way to tumor growth, angiogenesis, and immune evasion (202–205). For all these reasons, tumor-associated CKRs are considered suitable targets for cancer therapy (206). Nevertheless, generating antibodies against these Ags is particularly challenging due to, among other reasons, a complex and unstable native conformation (206, 207). So far, few anti-CKRs are under study in preclinical or early clinical phases and only one has been approved for clinical use (208).

### C-C-Motif-Chemokine Receptor 4 (CCR4)

Under homeostasis, this receptor and its ligands, the chemokines CCL17 and CCL22, predominantly contribute to the biology of Th2, Th17, Treg, and skin-homing T-cells positive for cutaneous lymphocyte antigen (CLA) (209–211). In addition, CCR4 has been implicated in the pathogenesis of inflammatory diseases and cancer, being overexpressed in several T-cell disorders including adult T-cell leukemia–lymphoma (ATL), peripheral T-cell lymphoma (PTCL), and cutaneous T-cell lymphoma (CTCL) (212–214).

In cancer therapy, mogamulizumab (KW-0761) is the first approved and clinically tested antibody against a CKR and, in addition, the first glycoengineered antibody to be marketed. This IgG1 antibody is directed to the N-terminal region of human CCR4. Despite this, it does not block the interaction between CCR4 and its ligands, thereby not interfering with CCR4-mediated protumor functions or migration (215, 216). Moreover, it does not bind complement molecules either. Nevertheless, its Fc was selectively defucosylated to reach a potent ADCC *via* high-affinity binding to the FcγRIIIa on effector cells (215, 217). As a result, phase I and II clinical trials investigating mogamulizumab in T-cell malignancies demonstrated its effectiveness and led to the approval for use in Japan for rrATL in 2012 and rrCTCL in 2014 (208, 218). Given the safety and efficacy of mogamulizumab, different clinical studies are underway for T-cell disorders (208, 219). In addition, based on preclinical evidence, studies are being conducted to establish whether other diseases could be targeted by mogamulizumab therapy, including certain NK-cell lymphoproliferative disorders (220) and Hodgkin’s lymphoma (HL) (221). Interestingly, in HL, the majority of the cells in TME are TAMs, Tregs, and CD4^+^ Th2 cells recruited by chemokines secreted by tumor cells such as CCL17 (222). This infiltrate probably enables tumors to escape from immune surveillance. Therefore, it is conceivable that targeting CCR4-positive cells in HL niche might revert this immunosuppresive environment enhancing the antitumor immunity. Indeed, in CTCL patients, a single dose of mogamulizumab decreased the fraction of CCR4-positive malignant T-cells, with a concomitant reduction of CCR4^+^ Tregs (223). Notably, similar effects on Treg subsets were observed in melanoma patients (224). All together, these results prompted phase I–II clinical studies in solid tumors not expressing CCR4 in order to evaluate the potential of mogamulizumab as immunomodulatory drug. Finally, the lack of neutralization of CCR4-ligands interaction by mogamulizumab leaves room to novel mAbs able to target this interaction. In preclinical phases, mAb1567 and its high-affinity variant (mAb2-3) were able to abolish CCR4-mediated chemotaxis of malignant cells and Tregs (225, 226). Moreover, *in vitro* studies confirmed that both antibodies mediated CDC and ADCC (225, 227), whereas the derivative mAb2-3 affected Treg functions and survival by means of CD25 shedding (226).

### C-X-C-Motif-Chemokine Receptor 4 (CXCR4)

This CKR and its chemokine CXCL12 (or stromal cell-derived factor 1α) regulate hematopoietic development, lymphoid tissue architecture, and hematopoietic cell trafficking. Additionally, this couple controls organogenesis and development in several tissues (228, 229), hence CXCR4 is not surprisingly overexpressed in more than 23 different human cancers and has been demonstrated to be particularly relevant in B-cell malignancies like B-ALL, CLL, or MM (230, 231), T-cell malignancies such as T-ALL (232), and myeloid malignancies like AML (233). In these conditions, CXCR4 causes tumor cell trafficking and homing into lymphoid and non-lymphoid tissues where CXCL12 is produced. Here, the couple CXCR4/CXCL12 keeps leukemic cells in close contact with stromal cells and extracellular matrix that together provide growth-promoting and antiapoptotic signals which facilitate resistance to chemotherapy and disease relapse (234–239). All together, these data strongly indicate that therapeutic strategies targeting the CXCL12–CXCR4 axis represent an attractive investigative approach to disrupt the leukemia–stromal interaction.

The first anti-CXCR4 clinically tested was ulocuplumab (BMS-936564), an IgG4 that blocks CXCL12 binding to its receptor thereby inhibiting CXCL12-induced migration and calcium flux (240). In this context, ulocuplumab is comparable to AMD3100 (Plerixafor-Mozobil), a small molecule CXCR4 inhibitor. However, ulocuplumab, but not AMD3100, induces caspase-independent apoptosis on a panel of cell lines and primary samples from AML, CLL, and MM patients (240–242). Both mechanisms contribute to the efficacy of ulocuplumab as monotherapy observed in xenograft models of the aforementioned diseases (240). The first clinical report on ulocuplumab suggests safe and significant antileukemia activity in AML patients, achieving fairly respectable complete remissions (CR/CRi) of 51%, and, notably in four patients, CR/CRi was documented after a single dose of ulocuplumab monotherapy (NCT01120457) (243). Results on other conditions are not available yet. Another IgG4 targeting CXCR4 is LY2624587, a humanized antibody deeply modified to eliminate half-antibody exchange associated with human IgG4 isotypes (244). Similar to ulocuplumab, LY2624587 inhibits CXCL12 binding to CXCR4 thus abrogating CXCR4-mediated survival and migration. The first clinical trial of LY2624587 (NCT01139788) was completed on 2011; however, results have not yet been published. Besides IgG4 isotypes, novel anti-CXCR4 antibodies with IgG1 isotype are demonstrating to be effective in preclinical phases. This is the case of hz515H7 (245) or PF-06747143. The latter was the first anti-CXCR4 mAb with an IgG1 scaffold to be evaluated in humans (NCT02954653), specifically in AML patients (246, 247). Like IgG4 formats, IgG1 antibodies are antagonist that block tumor cell chemotaxis toward CXCL12 and induce tumor cell apoptosis in either presence or absence of stromal cells (245, 246). In contrast, IgG1 isotypes trigger potent ADCC and CDC, which are involved in the antitumor effect observed in AML and CLL models as monotherapy or in combination with standard therapy (245–247). Currently, there is evidence suggesting that anti-CXCR4-IgG4 antibodies are generally safe although they induce short-term toxicity affecting the process of normal hematopoiesis with the result of myelosupression, or a deleterious effect on immune cells where CXCR4 is widely expressed (243). In addition, the off-target adverse event of hyperleukocytosis was reported in a number of patients. Finally, owing to the ubiquitous expression of CXCR4, long-term effects should be carefully evaluated, even more with the upcoming IgG1 molecules as they may trigger off-target ADCC or CDC.

### CCR2 and Others

CCR2 is another CKR targeted by an antibody under clinical development. Plozalizumab (MLN-1202) is a neutralizing antibody that showed a positive effect in phase II for the treatment of bone metastases (NCT01015560) (206). Interestingly, recent preclinical evidence suggests that targeting CCR2 may be effective in the setting of AML (248) and MM (249). MM cells from patients with bone lesions overexpress CCR2, while osteoclasts secrete chemokines that act as growth factors for tumor cells. In this scenario, targeting CCR2 could reduce MM cells survival and prevent drug resistance similar to CXCR4 antagonism (249). Many other CKRs with pathogenic role in hematological malignancies were preclinically validated as good targets for mAb-based therapy. This includes antibodies against CCR7 (34, 250) and CCR9 (251).

Recent evidence on a CXCL12-neutralizing RNA oligonucleotide reveals that targeting the chemokine instead of the CKR may interfere with CXCR4-mediated drug resistance in CLL and MM (252). These data support a rationale for clinical development of mAbs targeting chemokines instead of their corresponding receptors. However, to date no mAb targeting chemokines has been included in a clinical trial for cancer therapy. There are two plausible explanations. First, targeting chemokines does not activate the host immune response against tumor cells. Second, a cell surface-restricted receptor is more efficiently targeted than delocalized secreted chemokines (206, 207). Moreover, a recent study in cynomolgus monkeys demonstrated that targeting the chemokine CCL21 with a novel mAb (QBP359) requires impractical large doses and frequent administration to maintain suppression of CCL21 in the clinical setting. In other words, it is difficult to target soluble proteins with high synthesis rates, a common characteristic to many chemokines (253).

## Antibodies Targeting the Tumor Niche

Malignant cells are surrounded by different types of leukocytes and stromal cells that compose an extremely relevant source of soluble factors and adhesion molecules that promote tumor progression and escape from conventional treatments (254, 255). We have already referred in a separate section how clinical antibodies neutralize CKRs and their protumor activities in the TME. Below we review other approaches to disrupt the tumor niche, including: (i) neutralizing soluble survival/growth factors (mainly cytokines) or their associated receptors, all of them validated and valuable targets of antibody-based therapies of immunological disorders (256); (ii) blocking adhesion molecules that lodge tumor cells to their protective niche (257, 258); and (iii) blocking angiogenesis, an important process during development and vascular remodeling (259) that feeds tumor growth and progression (259, 260).

### Soluble Factors and Associated Receptors

#### B-Cell Activating Factor (BAFF) and A Proliferation Inducing Ligand (APRIL)

These TNF-family members are produced as type II transmembrane proteins that are then proteolytically cleaved and released in soluble form (261). BAFF and APRIL are produced by a variety of hematopoietic and non hematopoietic cells including stromal microvascular endothelial cells and osteoclasts. Both factors share two receptors: transmembrane activator and cyclophilin ligand interactor (TACI) and B-cell maturation antigen (BCMA; CD269). Additionally, BAFF binds strongly to BAFF receptor (BAFF-R) (261). These receptors have distinct expression patterns and mediate separate functions. BAFF-R is absent on B-cell precursors but is gained on immature B-cells upon acquiring a functional BCR, which is critical for survival and maturation of immature B-cells. TACI is expressed on memory B-cells and is necessary for T-independent responses and promotion of class switch recombination in B-cells. Last, BCMA expression is restricted to plasmablasts and plasma cells and promotes their long-lived survival (261–263).

BAFF and APRIL are particularly relevant in MM, where BCMA and both soluble factors are augmented in samples from patients compared to healthy donors, and ligand–receptor interactions lead to increased survival of malignant cells (264–267). Moreover, higher concentrations of APRIL may promote resistance to lenalidomide, bortezomib and other standard-of-care drugs, and also may drive expression of programmed cell death ligand 1 PD-L1, interleukin (IL)-10, and TGFβ on BCMA^+^ tumor cells creating an immunosuppressive niche that favors tumor cells (266). In recent years, compelling evidence has suggested that neutralizing APRIL or BAFF could diminish MM cell survival, revert the immunosuppressive phenotype on BCMA^+^ cells and reduce resistance of malignant cells to treatment, regardless of the presence of protective stromal cells (266, 268–270). Tabalumab (LY2127399), a human IgG4 mAb that neutralizes membrane-bound and soluble BAFF, was entered in clinical trials after demonstrating both antitumor activity and osteoclastogenesis inhibition in xenograft models of MM (270). Results in two different studies showed limited efficacy of tabalumab in combination with standard-of-care drugs (NCT00689507, NCT01602224) (271, 272). In the near future, new molecules will burst into the field such as BION-1301, an anti-APRIL neutralizing mAb able to fully suppress *in vitro* APRIL-induced B-cell IgA and IgG class switching (273).

Other antibodies block BAFF-R, including VAY736 and belimumab, or are aimed to deliver payloads to tumor cells expressing BCMA as exemplified by two novel inmmunoconjugates in clinical studies: AMG 224 and GSK2857916 (Table 6). Notably, GSK2857916 acts through multiple mechanisms. It specifically blocks cell growth *via* G2/M arrest, induces caspase 3-dependent apoptosis in MM cells, and strongly inhibits colony formation by MM cells. Furthermore, GSK2857916 recruits macrophages and mediates ADCP of MM cells (274). Finally, BI836909 and JNJ-6400795 are the first MM cell-specific BiTEs in development, and both target BCMA/CD3 (275). Clinical studies to evaluate safety and efficacy of both BiTEs are still recruiting MM patients (NCT02514239 and NCT03145181).

#### Receptor Activator of Nuclear Factor Kappa-B Ligand (RANKL)

This soluble member of the TNF family is a key mediator in the pathogenesis of a broad range of skeletal diseases since it binds to RANK on preosteoclasts and mature osteoclasts which are involved in bone resorption (276). In particular, malignant plasma cells produce RANKL leading to an imbalance between bone formation and resorption, local bone lysis and the development of osteolytic lesions in MM patients (277, 278). Denosumab is a human IgG2 antibody that binds to the soluble and cell membrane-bound forms of RANKL thus preventing RANK-mediated differentiation, activation, and survival of osteoclasts. As a consequence, bone resorption and bone destruction are reduced (279). As expected, and based on its MOA, denosumab is not effective reducing tumor burden neither improving responses in MM nor B-NHL. But denosumab does inhibit RANKL regardless of previous exposure to bisphosphonates, standard-of-care drug in bone lessions (280) and delays the time to the first on-study skeletal-related event with similar results to zoledronic acid, another standard-of-care treatment (NCT00330759) (281). Similar to IgG1, denosumab is a big molecule that, contrary to bisphosphonates, is not cleared by the kidneys. Therefore, current investigation (NCT02833610) is evaluating whether denosumab could fill the unmet need for bone-targeted therapies in MM patients with renal insufficiency (approximately 25–50% of all patients) (282). Finally, denosumab has demonstrated efficacy at solving bisphosphonate-refractory hypercalcemia in hematological cancers (NCT00896454) (283). Despite the current US FDA-approved label for denosumab it does not include MM nor NHL, this situation may be reverted depending on the forthcoming results from these studies.

#### Interleukin-6

IL-6 is a pleiotropic cytokine with a critical role in the pathogenesis of MM and B-NHL by promoting tumor cell growth and interfering with chemotherapy drugs (284). Different mAbs against IL-6 or its soluble receptor IL-6R have been developed, with the two most promising being the chimeric siltuximab (CNTO 328) that neutralizes the cytokine, and tocilizumab that blocks the receptor (285). Siltuximab, was recently registered for multicentric Castleman’s disease and evaluated as a single agent or in combination with other agents in advanced MM and B-NHL (particularly CLL). Again, mAb-based strategies targeting soluble factors produce discouraging results in hematological malignancies. In addition, results are modest probably due to the complex interaction between malignant clones, inflammatory background and host response (NCT00412321, NCT00911859, NCT00401843) (286, 287). However, new investigations aim to uncover the application of siltuximab in the treatment of Waldenström macroglobulinemia and the early phase of smoldering MM (285). Moreover, one trial is exploring the utility of blocking the IL-6R in CLL (NCT02336048).

#### IL-3 Receptor Alpha Chain (IL-3Rα; CD123)

Interleukin-3 stimulates cell cycle progression in early hematopoietic progenitors and enhances the differentiation of various hematopoietic cells while inhibiting apoptosis (288). IL-3Rα is a novel molecular target that has emerged as a highly specific entity for CML, AML blasts, and LSCs (289–291). Notably, normal hematopoietic stem cells have limited expression of CD123 (289, 292). One of the first humanized anti-IL-3Rα antibodies tested in clinical trials was CSL360, a chimeric IgG1 molecule that achieved an improvement in blasts and LSCs percentage in bone marrow, but no clinical responses in high-risk rrAML (NCT00401739) (293). These results showed that the blockade of IL-3Rα alone was ineffective, leading to the development of second-generation molecules able to kill IL-3Rα-positive tumor cells by means of immune effector mechanisms. The one in most advanced stages is talacotuzumab (JNJ-56022473/CSL362), an Fc-engineered derivative from CSL360 which is undergoing phase II–III studies for rrAML (NCT02992860, NCT02472145). Talacotuzumab induces potent *in vitro* ADCC against IL-3Rα-expressing AML blasts/LSC and reduces leukemic cell growth in murine xenograft models of human AML (294). In addition, talacotuzumab inhibits IL-3–stimulated rescue of tyrosine kinases inhibitors (TKIs)-induced cell death, demonstrating that resistance to previous standard-of-care could be reverted (295). Actually, the combination of TKI therapy and talacotuzumab may eliminate leukemic cells *in vivo* more effectively than TKI treatment alone (296). Another second-generation mAb with similar MOA is XmAb14045 that will be tested early in a phase I study (NCT02730312). With different MOA, KHK2823 is a novel non-fucosylated fully human IgG1 mAb which mediates ADCC without inducing CDC. Its safety and efficacy is under evaluation in a phase I study (NCT02181699).

Other approaches targeting CD123 are based on bi-specific platforms. Examples are JNJ-63709178 (NCT02715011), a humanized CD123xCD3 DuoBody and flotetuzumab (NCT02152956), a CD123xCD3 bi-specific antibody-based molecular construct named dual affinity retargeting (DART) molecule. Both bispecific antibodies are effective *in vitro* and *in vivo* in preclinical settings (297). Nevertheless, recent evidence on human CD123-redirected T-cells (CAR-T123) shows some concerns regarding toxicity related to off-target events (298), suggesting the possibility that the same effects could be found with the redirection of T-cells by bi-specific antibodies. Finally, owing to CD123 internalization upon mAb binding, novel immunoconjugates aim to target CD123 (Table 6).

#### IL-2 Receptor Alpha Chain (IL-2Rα; CD25)

Commonly expressed by activated T- and B-cells, some thymocytes and myeloid precursors, IL2-Rα is also found in most of the malignacies corresponding to such lineages, particularly in ATL where IL2-Rα functions as the receptor for human T-cell leukemia virus 1 (HTLV-1) (299). Few mAbs have been developed for T-cell neoplasia. One of them, the chimeric IgG1 basiliximab selectively blocks IL-2Rα, thereby preventing IL-2-mediated activation of lymphocytes. Nevertheless, it lacked of activity in patients (299). Another anti-IL-2Rα is daclizumab, a humanized antibody which shows potential in T-cell disorders and HL, although its activity needs to be confirmed in a big cohort of patients (300, 301). A likely explanation for the modest results of anti-IL-2R therapy is related to the pharmacokinetics/pharmacodynamics of daclizumab. Indeed, a phase I–II study suggested that higher doses than previously used may be required to achieve clinical responses since high doses were needed to saturate targets in extravascular sites (301). Low activity in phase I–II was also documented for LMB-2, an immunotoxin comprised of the Fv of an anti-CD25 mAb connected to an exotoxin (302). The limited efficacy of naked or toxin-conjugated antibodies has led to the conjugation of basiliximab and daclizumab with radionuclides. Currently, both molecules are under evaluation. Interestingly, in HL daclizumab linked to radionuclides shows efficacy in patients with tumor cells expressing IL-2R, and in patients whose tumor cells lacking the receptor, suggesting off-target effects on accessory cells (303). Based on the same rationale, both basiliximab and daclizumab are being explored as adjuvant therapy to eliminate IL-2-Rα-positive Tregs in MM or to eliminate IL-2-Rα-positive naive T-cells to prevent the development of graft-versus host disease.

#### Type I Insulin-Like Growth Factor Receptor (IGF-1R; CD221)

This ubiquitously expressed tetramer binds insulin growth factor 1 (IGF-1) to activate multiple signaling pathways involved in cell growth, differentiation, migration, and cell survival (304). IGF-1R also mediates anchorage-independent growth and survival, and migration, thus facilitating tumor establishment and progression (305–307). IGF-1R has been widely studied in hematological tumors where a pathogenic role has been found, among others, for myeloid leukemias (308), and several B-cell malignancies (304, 309) but an exceptional role has been uncovered for MM (310). Therefore, the therapeutic potential of IGF-1R has been explored in MM with three different mAbs that directly block IGF-1R: dalotuzumab, AVE1642, and figitumumab. All of them prevent the binding of IGF-1 and the subsequent activation of PI3K/AKT signal transduction, nonetheless results derived from clinical studies were disappointing (310). In phase I studies, only dalotuzumab showed an evaluable antimyeloma activity (NCT00701103) (311). In contrast, AVE1642 (NCT01233895) and figitumumab (NCT01536145) did not result in significant improvement as single agents or in combination with standard-of-care drugs (312, 313). Hence, no further evaluation of these mAbs in MM patients is currently ongoing and the development of dalotuzumab in MM was consequently also discontinued. These discouraging results could be explained by the emergence of tumor cell independence from their microenvironment due to intraclonal heterogeneity, the involvement of other growth factors and the existence of hybrid receptors composed of IGF-1R and 2R that can be activated by all IGF ligands (314). Anti-IGF-1R mAbs are unable to neutralize these hybrid receptors. Moreover, it is thought that circulating IGF-1R can interact with the IGF-1R targeting antibodies and prevent their interaction with the IGF-1R on cancer cells (310).

#### Granulocyte-Macrophage Colony-Stimulating Factor (GM-CSF; CSF2)

This cytokine is a monomeric glycoprotein secreted by immune cells, endothelial cells and fibroblasts. Overall, GM-CSF participates in the development of innate immune cells since it stimulates stem cells to produce granulocytes and monocytes (315). GM-CSF is also involved in the pathogenesis of chronic myelomonocytic leukemia (CMML), where progenitor expansion through STAT5 signaling is mediated (316). Based on this, lenzilumab (KB003), a humanized antibody formerly developed for inflammation, is being investigated as single agent in CMML (NCT02546284) (317). In primary samples from CMML patients, lentizumab bound the cytokine and directly interrupted binding to its cognate receptor inducing apoptosis *in vitro* (318). However, the curative application in humans is uncertain as some CD34-positive subsets, including the LSCs, seem to be insensitive to GM-CSF signaling (318). Results from the ongoing clinical study will shed some light on the subject.

#### Hepatocyte Growth Factor (HGF)

This soluble factor is the only known ligand for the c-Met receptor tyrosine kinase that when bound to HGF activates key oncogenic signaling pathways that increase cell proliferation, survival, migration and invasion in several human cancers, including MM where HGF expression predicts poor prognosis (319). Ficlatuzumab (AV-299) is a potent HGF-neutralizing mAb able to interrupt HGF/c-Met interaction thus inhibiting c-Met-induced phosphorylation, cell proliferation, cell invasion and cell migration. With proven efficacy in solid tumors (320), a phase I study aimed to examine its efficacy in MM and NHL (NCT00725634), although preliminary results indicate that clinical effects are only seen in MM (321).

### Adhesion Molecules

#### CD44

This cell-surface glycoprotein is a receptor for hyaluronic acid (HA), osteopontin, collagen, and matrix metalloproteases, which are typically found in the microenvironment of BM and lymphoid tissues (257). CD44 is particularly expressed by AML-LSCs and CLL cells which take advantage of HA-CD44 signaling to promote leukemic survival *via* PI3K/AKT and MAPK/ERK pathways (257, 322). Since AML-LSCs are more dependent on CD44 for their anchoring in the BM niche than their normal counterparts, CD44 is an exciting target to mobilize leukemic cells out of their protective niche (257). A novel humanized neutralizing mAb, RG7356 (RO5429083), has recently been evaluated alone or in combination with cytarabine in a phase I trial in rrAML patients (NCT01641250). RG7356 does not activate effector cells or complement. Very limited activity was observed in this study although the mAb was well tolerated (323). The ability of CD44 to complex with different partners, overcoming the neutralization mediated by the antibody, may explain this outcome. In CLL, the expression of CD44, in cooperation with VLA-4 and MMP9, helps in creating a protective TME within the lymphoid organs that circumvents spontaneous and drug-induced apoptosis in CLL cells (324). In preclinical models of CLL, RG7356 provoked apoptosis of CLL cells in a caspase-dependent manner and regardless of the presence of protective co-cultured stromal cells or HA, or even regardless of BCR signaling (325). These results indicate that RG7356 might have therapeutic activity in CLL patients.

#### Very Late Antigen 4 (VLA4; CD49d)

This molecule is the α-chain of the α4β1 integrin heterodimer which is normally expressed on monocytes and lymphocytes cell surface (326, 327). VLA-4 is involved in the firm adhesion step during the extravasation process, mediating the binding to fibronectin or to vascular cell adhesion molecule-1 (VCAM-1) located on the surface of endothelial cells (326, 327). In several hematological malignancies CD49d is considered as one of the main players at the TME as it mediates both cell–cell and cell–matrix interactions delivering prosurvival signals and protecting tumor cells from drug-induced damage (258, 328–330). Despite this, no anti-VLA4 antibodies are under development for blood cancers. In this context, the recombinant IgG4 anti-CD49d antibody natalizumab, which is an FDA-approved drug for relapsing multiple sclerosis, has demonstrated the potential to revert chemo-sensitivity and to inhibit both *in vitro* and *in vivo* adhesion of MM cells to non-cellular and cellular components of the TME. It also has the potential to arrest tumor growth in a xenograft model of MM (329). Unfortunately, a trial evaluating natalizumab as a single agent in MM patients was terminated due to low enrollment (NCT00675428). Natalizumab is also able to restore drug sensitivity in B-cell lymphomas (328) and primary ALL (330) providing the rationale for the clinical evaluation of natalizumab in many hematological tumors, preferably in combination with novel agents to enhance tumor cell cytotoxicity and improve patient outcome (329). Nevertheless, it should be taken into account that natalizumab treatment is associated with appearance of progressive multifocal leukoencephalopathy which may, in turn, dissuade this approach in hematological malignancies (331).

### Angiogenesis

#### Vascular and Endothelial Growth Factor (VEGF)

This molecule is one of the most important mediators of neo-angiogenesis and tumor growth (332). Out of the five members of the VEGF family described to date, VEGF-A and its receptor VEGFR-2 are the main targets of current antiangiogenic agents (332). Bevacizumab is an IgG1 antibody that binds to all isoforms of VEGF-A preventing the interaction with its receptors and their subsequent activation (333). In solid tumor, this antibody promotes a regression of immature tumor vasculature, normalization of remaining tumor vasculature and inhibition of further tumor angiogenesis (334). In hematological malignancies, bevacizumab has been tested as a tool to solve resistances to previous chemotherapies. In myeloid malignancies, bevacizumab has not worked as monotherapy or combined with standard therapies (335). In CLL, preclinical studies demonstrated that bevacizumab was a proapoptotic and antiangiogenic drug (336, 337). Despite having no significant clinical activity as monotherapy (NCT00290810) (338), in combination with chemotherapy regimens the results had a better outcome (339). In FL, a phase II study (NCT00193492) revealed that a combination of rituximab with bevacizumab significantly extended progression free survival, although bevacizumab increased the toxicity as well (340). Encouraged by these works, bevacizumab has been used as adjuvant therapy in many other B-cell disorders, where addition of the anti-VEGF did not show improvement of the therapeutic responses (341, 342). It is tempting to assume that anti-VEGF in combination with other chemoimmunotherapies is a promising therapy for CLL and FL patients, but a close follow-up is recommended to ascertain the potential toxicities, including left ventricular dysfunction and heart failure, observed in many of the cited studies.

#### Endosialin (CD248; TEM1)

This glycoprotein is selectively expressed in vascular endothelial cells of malignant tumors (343). Targeting endosialin showed antitumor activity in different preclinical models where endosialin function was suppressed with the antiangiogenic antibody MORAB-004 (ontuxizumab) (343). Although the MOA of this drug is not completely understood, it was suggested that endosialin is removed from the cell surface by means of MORAB-004–mediated internalization. MORAB-004 could affect cellular signaling as well as protein–protein interactions that serve to communicate signals in the TME between tumor and stromal cells. Additional work is under way to further establish the exact mechanism of action of MORAB-004 (343). MORAB-004 antitumor activity has been observed in several phase I studies on solid tumors and phase II studies have recently started. Despite the role of endosialin in blood cancers is not fully understood, patients with different blood disorders have been enrolled in a phase I study (NCT01748721), although no results have been published (344).

## Antibodies Targeting Immune Checkpoints

Antitumor innate and adaptive immune responses rely on cell-activation and cell-exhaustion balances which are regulated by stimulatory or inhibitory molecules most belonging to the B7-CD28 and the TNF-TNFR superfamilies (345). Due to their function modulating immune responses, these NLSAs are commonly known as “immune checkpoints.” Although checkpoint targeting with specific antibodies is a relatively new area (346), their accessibility to cell membranes and significance in regulating immune responses made them a very attractive therapy option, as exemplified by the plethora of novel agents that have been already approved or are under intensive studies in solid tumors. In Table 4, an account of the landscape of immune-checkpoint regulators in hematological malignancies is provided. Commonly, immune-checkpoints do not target tumor cells directly, but instead act on lymphocytes to boost their endogenous antitumor activity reversing tumor immune escape (347, 348). It is not our intention to review these types of molecules. Instead, in the next section, we will analyze a second-generation of immunomodulatory antibodies targeting receptors expressed in both tumor and immune cells. These antibodies are armed with a dual MOA combining direct tumoricidal properties with the ability to restore host antitumor immunity.

**Table 4:** Characteristics of antibodies directed to immune checkpoint receptors.

Target/expression	mAb (commercial name/originator)	IgG class	MOA	Active indications in HMs (highest phase)	References
**Inhibitory receptors**							
Programmed death-1 (PD-1)/T-cells, NK cells, NKT cells, Treg and B-cells	Nivolumab, BMS-936558, MDX-1106 (*Opdivo*/Medarex; Ono Pharmaceutical)	Fh IgG4	Blocks binding of PD-1 to PD-L1 and PD-L2 thus enhancing anti-tumor immunity.	Approved (HL)	(83–88)
				III (MM/NCT02726581 ^a^R)	
				II–III (AML/NCT02275533 ^b^R)	
				II (CLL/NCT 02420912 ^a^R)	
				II (DLBCL/NCT02038933 ^b^ANR)	
				II (FL/NCT02038946 ^b^ANR)	
				I–II (B-NHL, T-NHL/NCT02985554 ^b^R)	
				I (CML/NCT02011945 ^a^ANR)	
				I (FL/NCT03245021 ^a^NYR)	
				I (B-ALL/NCT02819804 ^a^R)	

	Pembrolizumab, MK-3475 (*Keytruda*/Merck & Co; The Leukemia & Lymphoma Society)	Hz IgG4	Blocks binding of PD-1 to PD-L1 and PD-L2 thus enhancing anti-tumor immunity.	III (HL/NCT02684292^b^ R)	
				II–III (MM/NCT02906332 ^a^ANR)	
				II (DLBCL/NCT02362997 ^b^R)	
				II (FL/NCT02446457 ^a^R)	
				II (T-NHL, NK-L/NCT03021057 ^b^ANR)	
				II (ALL/NCT02767934^b^ R)	
				II (AML/NCT02768792 ^b^R)	
				II (MF/SS/NCT02243579 ^b^ANR)	
				II (CLL/NCT02332980 ^a^R)	
				I–II (MCL/NCT03153202 ^a^R)	

	Pidilizumab; CT-011, MDV9300 (CureTech)	Hz IgG4	Blocks binding of PD-1 to PD-L1 and PD-L2 thus enhancing anti-tumor immunity.	II (MM/NCT01067287 ^a^ANR)	
				II (FL/NCT00904722 ^a^C)	
				II (DLBCL/NCT02530125 ^b^ANR)	
				II (AML/NCT01096602 ^a^ANR)	
				I–II (MM/NCT02077959 ^a^ANR)	

	MEDI0680, AMP-514 (Amplimmune)	Hz IgG4	Blocks binding of PD-1 to PD-L1 and PD-L2 thus enhancing anti-tumor immunity.	I–II (B-NHL, DLBCL/NCT02271945 ^a^C)	

	REGN2810 (Regeneron Pharmaceuticals)	Fh IgG4	Blocks binding of PD-1 to PD-L1 and PD-L2 thus enhancing anti-tumor immunity.	I–II (B-NHL, HL/NCT02651662 ^a^R)	
				I–II (MM/NCT03194867 ^a^NYR)	

	PDR001 (Novartis)	Hz IgG4	Blocks binding of PD-1 to PD-L1 and PD-L2 thus enhancing anti-tumor immunity.	II (DLBCL/NCT03207867 ^a^NYR)	
				I (AML/NCT03066648 ^a^R)	
				I (MM/NCT03111992 ^a^R)	

	BGB-A317 (BeiGene)	Hz IgG4	Blocks binding of PD-1 to PD-L1 and PD-L2 thus enhancing anti-tumor immunity.	II (HL/NCT03209973 ^b^R)	
				I (B-NHL/NCT02795182 ^a^R)	

	SHR-1210 (Jiangsu Hengrui Medicine Co.)	Fh IgG4	Blocks binding of PD-1 to PD-L1 and PD-L2 thus enhancing anti-tumor immunity.	II (HL/NCT03155425 ^b^NYR)	

	Js001 (Shanghai Junshi Biosciences)	Hz mAb	Blocks binding of PD-1 to PD-L1 and PD-L2 thus enhancing anti-tumor immunity.	I (Lymphoma/NCT02836834 ^b^R)	

PD-L1 (CD274 or B7-H1)/tumor cells	Atezolizumab, MPDL-3280A, RG7446 (*Tecentriq*/Genentech)	Hz IgG1	Glyco-Fc	Blocks PD-L1/PD-1 interaction thus enhancing antitumor immunity.	(89–91)
				II (HL/NCT03120676 ^b^R)	
				II (CLL/NCT02846623 ^a^R)	
				I–II (DLBCL/NCT02926833 ^a^R)	
				I–II (AML/NCT02935361 ^a^R)	
				I–II (CML/NCT02935361^a^R)	
				I (FL/NCT02220842 ^a^R)	
				I (MM/NCT02784483 ^b^R)	

	BMS-936559, MDX-1105 (Medarex)	Fh IgG4	Blocks PD-L1/PD-1 interaction thus enhancing antitumor immunity.	I (HL, NHL, CML, MM/NCT01452334 ^b^W)	

	Durvalumab, MEDI-4736 (Medimmune)	Fh IgG1	Blocks PD-L1/PD-1 interaction thus enhancing antitumor immunity.	II (AML/NCT02775903 ^a^R)	
				II (DLBCL/NCT03003520 ^a^R)	
		Glyco-Fc	ADCC		
				II (MM/NCT03000452 ^a^R)	
				II (NK-T Lymphoma/NCT03054532 ^a^NYR)	
				I–II (CLL/NCT02733042 ^a^R)	
				I–II (NK-T Lymphoma/NCT02556463 ^a^R)	
				I–II (FL/NCT02401048 ^a^ANR)	

	Avelumab, MSB0010718C (*Bavencio*/EMD Serono; Merck KGaA)	Fh IgG1	Blocks PD-L1/PD-1 interaction thus enhancing antitumor immunity.	III (DLBCL/NCT02951156 ^a^ R)	
				II (T-NHL/NCT03046953 ^b^ NYR)	
				I–II (AML/NCT02953561 ^a^ R)	
				I–II (B-NHL/NCT03169790 ^a^ NYR)	
				I (HL/NCT02603419 ^b^R)	

CTLA-4 (Cytotoxic T lymphocyte-associated antigen 4, CD152)/T-cells, Treg, NK cells	Ipilimumab (*Yervoy*/Medarex)|	Fh IgG1	Blocks the interaction of CTLA-4 with CD80/CD86 thus enhancing antitumor immunity.	II (AML/NCT02397720 ^a^R)	(83, 92, 93)
				I–II (HL/NCT01896999 ^a^R)	
				I–II (MM/NCT02681302 ^a^R)	
				I (CML/NCT00732186 ^a^W)	
				I (B-NHL/NCT01729806 ^a^ANR)	
				I (B-ALL/NCT02879695 ^b^R)	

	Tremelimumab, CP-675,206 (Pfizer)	Fh IgG2	Blocks the interaction of CTLA-4 with CD80/CD86 thus enhancing antitumor immunity.	I–II (B-NHL/NCT02205333 ^a^T)	
				I (DLBCL/NCT02549651 ^a^R)	
				I (MM/NCT02716805 ^a^R)	

KIR2D (killer inhibitory receptor 2D)/NK cells	Lirilumab, IPH2102 (Innate Pharma, Novo Nordisk)	Fh IgG4	Blocks the interacion of HLAC with KIR2D thus augmenting NK-cell activity.	II (AML/NCT01687387 ^b^C) disc.	(94, 95)
				II (CLL/NCT02481297 ^a^ANR)	
				I (MM/NCT02252263 ^a^ANR)	

NKG2A (CD94)/NK cells, CTLs	Monalizumab, IPH2201 (Innate Pharma; Novo Nordisk)	Hz IgG4	Blocks the interaction of HLA-E with NKG2A thus augmenting NK cells and CTLs reactivity.	I–II (CLL/NCT02557516 ^a^R)	(96, 97)
				I (HMs/NCT02921685 ^a^R)	

KIR3DL2 (killer inhibitory receptor 3DL2; CD158k)/Tumor cells	IPH4102 (University of Genoa/Innate Pharma)	Hz IgG1	ADCC	I (CTCL/NCT02593045 ^b^R)	(98)
			ADCP		
			Blocks KIR3DL2.		

LAG3 (lymphocyte-activated gene-3, CD223)/Th cells, Treg	BMS-986016 (Bristol-Myers Squibb)	Fh IgG4	Blocks the binding of LAG3 to MHC-II thus decreasing tumor suppressive activity.	I/II (HL/NCT02061761 ^a^R)	(99)
				I/II (DLBCL/NCT02061761 ^a^R)	
				I (CLL, MM/NCT02061761 ^b^R)	

TIM-3 (T-cell immunoglobulin- and mucin-domain-containing molecule 3)/T-cells, NK cells, monocytes	MBG453 (Novartis Pharmaceuticals)	Hz IgG4	Blocks TIM-3/Galectin-9 interaction thus enhancing Th1 responses and abrogating Treg suppressive functions.	I (AML/NCT03066648 ^a^R)	(100)

CD200 (OX-2)/Tumor cells	Samalizumab, ALXN6000 (Alexion Pharmaceuticals)	Hz IgG2/4	Blocks CD200/CD200R interactions, restoring CTLs functions and antitumor immunity.	I–II (AML/NCT03013998 ^a^R)	(101)
				I–II (CLL and MM/NCT00648739 ^a^C) disc.	

**Costimulatory receptors**					

CD137 (4-1BB)/T-cells, Treg, DCs, NK cells, NKT cells	Urelumab, BMS-663513 (Medarex)	Fh IgG4	Mimicks activation of CD137 mediated by CD137L (4-1BBL) inducing CTLs and NK cells activation.	II (CLL/NCT02420938 ^a^W)	(102–104)
				I–II (B-NHL/NCT02253992 ^a^R)	
				I (B-NHL/NCT01471210 ^b^C)	
				I (MM/NCT02252263 ^a^ANR)	

	Utomilumab, PF-05082566 (Pfizer)	Fh IgG2	Mimicks activation of CD137 mediated by CD137L (4-1BBL) inducing CTLs and NK cells activation.	III (DLBCL/NCT02951156 ^a^R)	
				I (FL/NCT01307267 ^a^R)	

OX40 (CD134)/T-cells, Treg	MEDI6469 (AgonOx; Providence Cancer Center)	m IgG1	OX40 triggering stimulates T-cells and blocks/depletes Treg	I–II (B-cell lymphomas/NCT02205333 ^a^T)	(105)
				Replaced by Tavolixizumab (MEDI0562)	

CD27/T-cells, B-cells, NKs	Varlilumab, CDX-1127 (Celldex Therapeutics Inc.)	Fh IgG1	Mimicks CD27-CD70 interactions which accelerate NK-mediated tumor clearance while generating an adaptive immune response	II (DLBCL/NCT03038672 ^a^NYR)	(106)
				I (B-NHL/T-NHL/NCT01460134 ^b^ANR)	

CD70/T-cells, B-cells, mDC, tumor cells	ARGX-110 (arGEN-X)	Hz IgG1	ADCC	I–II (AML/MDS/NCT03030612 ^a^R)	(107, 108)
		Glyco-Fc	ADCP		
			CDC		
			Blocks proliferation/survival of malignant cells.		
			Innhibits activation/proliferation of CD27-positive T-reg.		

CD80 (B7-1)/APCs, tumor cells	Galiximab, IDEC-114 (Biogen Idec)	Pz IgG1	ADCC	III (FL/NCT00363636 ^a^T, NCT00384150 ^a^T) disc.	(109, 110)
			PCD	II (B-NHL/NCT00516217 ^b^C) disc.	
			Inhibition of CD80 signaling		

CD40/APCs tumor cells	Lucatumumab, CHIR-12.12, HCD-122 (Novartis; XOMA)	Fh IgG1	ADCC	II (MM/NCT00231166 ^b^C) disc.	(111–115)
				I–II (NCT00670592 ^a^C) disc.	
			Antagonizes CD40L-mediated proliferation and survival		
				I (CLL/NCT00108108 ^a^T) disc.	
				I (FL/NCT01275209 ^a^C) disc.	

	Dacetuzumab, SGN-40 (Seattle Genetics)	Hz IgG1	ADCC, ADCP	II (DLBCL/NCT00435916 ^b^C, NCT00529503^a^C)	
			Partial agonist that triggers both cellular proliferation and activation in APCs which subsequently activate B-cells and T-cells		
				I–II (CLL/NCT00283101 ^b^C)	
				I (MM/NCT00079716 ^b^ C, NCT00664898^a^C)	

	SEA-CD40, SEA-1C10 (Seattle Genetics)	Hz IgG1	ADCC	I (B-NHLs, HL/NCT02376699 ^a^ R)	
	Derived from dacetuzumab	Glyco-Fc	Agonist that triggers both cellular proliferation and activation in APCs which subsequently activate B-cells and T-cells		

	ChiLob 7/4 (University of Southampton)	Ch IgG1	CDC, ADCC	I (DLBCL/NCT01561911 ^b^C)	
			Agonist that triggers both cellular proliferation and activation in APCs which subsequently activate B-cells and T-cells		

GITR (glucocorticoid-induced tumor necrosis factor receptor family-related gene)/NK cells, Th cells, CTLs, B-cells, APCs, Treg	GWN323 (Novartis)	Fh IgG1	Agonist that induces signaling through GITR abrogating Treg mediated suppression and enhancing Th, CTLs and NK cells proliferation and activation	I (B-cell lymphomas/NCT02740270 ^a^ R)	(116, 117)
		ADCC			

CD47/phagocytes and DCs	Hu5F9-G4 (Stanford University)	Hz IgG4	Blocks CD47	I–II (B-NHL/NCT02953509 ^a^R)	(118, 119)
				I (AML,MDS/NCT02678338 ^b^R, NCT03248479 ^b^NYR)	

	CC-90002, INBRX-103 (Inhibrx)	Hz IgG	Blocks CD47	I (AML/MDS/NCT02641002 ^b^R)	
			ADCP?		
				I (B-NHL/NCT02367196 ^a^R)	

*Antibodies that reached clinical studies*.

*^a^Combined therapy*.

*^b^Monotherapy*.

*mAb, monoclonal antibody; MOA, mechanisms of action; HMs, hematological malignancies; Ch, human–mouse chimeric; Fh, fully human; Hz, humanized; Pz, Human-primate chimeric; m, mouse; Glyco-Fc, glycoengineered Fc fragment; CDC, complement-dependent cytotoxicity; ADCC, antibody-dependent cell-mediated cytotoxicity; ADCP, antibody-dependent cell-mediated phagoytosis; PCD, programmed cell death; HL, Hodgkin’s lymphoma MM, multiple myeloma; AML, acute myeloid leukemia; CLL, chronic lymphocytic leukemia; DLBCL, diffuse large B-cell lymphoma; FL, follicular lymphoma; B-NHL, B-cell non-Hodgkin’s lymphoma; T-NHL, T-cell non-Hodgkin’s lymphoma; CML, chronic myeloid leukemia; B-ALL, B-cell acute lymphoblastic leukemia; NK-L, NK-cells lymphoma; MF, mycosis fungoides; SS, Sézary syndrome; MCL, mantle cell lymphoma; CTCL, cutaneous T-cell lymphoma; MDS, myelodysplastic syndrome; Treg, regulatory T-cells; Th, T helper cells; CTLs, cytotoxic T-cell lymphocytes; APCs, antigen presenting cells; DC, dendritic cells; disc., discontinued in hematological malignancies; NCT, number of clinical trial (clinicaltrials.gov); C, completed; R, recruiting; T, terminated; ANR, active non-recruiting; NYR, not yet recruiting; T, terminated; S, suspended; W, withdrawn*.

### PD-L1 (B7-H1; CD274)

Programmed death-1 (PD-1) is an inhibitory receptor member of the B7 receptor family with a significant role in immune regulation (349). PD-1 is upregulated on activated T-cells, NK cells, NKT cells, and B-cells among other leukocytes (349–351). In many types of cancers, PD-1 engagement may represent one means by which tumors evade immune surveillance and clearance (349). Cancer cells express PD-L1, a PD-1 ligand that upon binding to PD-1 on tumor-infiltrating lymphocytes (TILs) leads to impairment of antitumor responses through multiple mechanisms including inhibition of T-cell activation and proliferation (352, 353) and increase in T-cell apoptosis (349, 354). In addition, PD-L1 drives the differentiation of naive CD4^+^ T-cells into induced Tregs, which also express PD-1 on their cell surface, and maintain their suppressive function (355). The end result of the PD1 axis activation is T-cell exhaustion or anergy, dampening effector T-cell functions and leading to immune tolerance.

This situation prompted the development of different mAbs to target either the receptor or the ligand with the goal of “releasing the brakes” on effector T-cells preventing suppression of the antitumor response and causing tumor cytotoxicity. Similar to antibodies targeting PD-1 (nivolumab and pembrolizumab are the most widely marketed anti-PD-1 antibodies), anti-PD-L1 antagonists aim to restore effector T-cell and NKs activities while abrogating intra-tumoral Treg-mediated suppression (83). In addition, some anti-PD-L1 are able to mediate ADCC and other Fc-mediated functions. Four PD-L1 mAbs have demonstrated clinical activity in several solid tumors including atezolizumab, durvalumab, avelumab, and MDX-1105 (BMS-936559) (356). Activity of the IgG4 MDX-1105 and the low-ADCC inducer, Fc-engineered, humanized, IgG1 atezolizumab rely on blocking PD-L1. In contrast, durvalumab and avelumab combine two MOA: blocking PD-L1/PD-1 interactions, and directly killing PD-L1-positive tumor cells (356). Clinical studies involving both molecules are recruiting patients or just initiating in different hematological malignancies. Some of the diseases that could be targeted by anti-PD-L1 double MOA are: HL, B-ALL, FL, or MM (357–359). Nonetheless, anti-PD1 targeting in MM had low efficacy and, most notably, established MM therapies such as IMiDs are able to reduce PD-L1 on MM cells and could interfere with the outcome (360).

### CD40

This glycoprotein is a member of the TNFR superfamily that is principally expressed on APCs, but also on several tumors, such as B-cell lymphomas and carcinomas (111). Through the binding to its ligand CD40L (or CD154) on CD4^+^ T-cells, CD40 plays a key role in a broad variety of immune and inflammatory responses, including T-cell-dependent immunoglobulin class switching, memory B-cell development, germinal center formation, functional maturation of DC, and upregulation of macrophage cytotoxic function. To date, different anti-CD40 mAbs have been developed including three agonistic and one antagonistic which are being investigated in a range of lymphoid and solid tumors (111).

Lucatumumab (HCD122/CHIR12.12) is a fully human antibody that antagonizes CD40L-mediated proliferation and survival on CLL and MM cells, and triggers ADCC (111, 112, 361). Lucatumumab has overall modest activity as single agent or in combined regimens in multiple clinical studies on B-cell tumors, including HL (NCT00283101) (362), CLL (NCT00108108) (363), and MM (NCT00231166) (364). Similar to lucatumumab, the humanized IgG1 dacetuzumab (SGN-40) has tumoricidial activities in cultured NHL cells through ADCC, ADCP and direct apoptosis *via* caspase-3 activation. In contrast to lucatumumab, dacetuzumab does not prevent CD40/CD40L interaction, and behaves as a partial agonist by augmenting effector CTL responses (365, 366). The efficacy and safety of dacetuzumab as a single agent to treat rrMM, rrNHL, or rrCLL was investigated in three phase I studies, respectively (NCT 00079716, NCT00103779, NCT00283101), which demonstrated mild side effects but modest efficacy across the cancers tested (367–369). Nevertheless, combining dacetuzumab with chemotherapy and/or rituximab demonstrated synergistic activities in both preclinical and phase I clinical studies in rrDLBCL (NCT00529503, NCT00655837) (370–372).

Despite the limited activity of anti-CD40 antagonists, results with the partial agonist dacetuzumab and compelling evidence in preclinical models confirmed that CD40 agonists acting as CD40L could be a better venue to drive stronger antitumor responses (113, 373). Currently, two types of agonist anti-CD40 are available. The first type combines the activation of tumor-specific immune responses with a direct tumoricidal activity. In this group, we can include dacetuzumab along with the chimeric ChiLob7/4 and the human sugar-engineered SEA-CD40 antibodies (113–115, 365, 366). Upon binding to CD40, these drugs trigger both cellular proliferation and activation of APCs which activate innate and adaptive antitumor immunity (113, 373). In addition, these antibodies also directly kill CD40-expressing cancer cells through ADCC, and eventually inhibit proliferation and growth of CD40-expressing tumor cells. ChiLob7/4 has completed a phase I study in B-NHL showing a well-tolerated range of doses whereas SEA-CD40 is enrolling patients, at the time of writing, in a first phase I study in combination with pembrolizumab in solid tumors, B-NHLs and HL (NCT02376699).

A second type of CD40-directed antibodies triggers antitumor immune responses as sole MOA. Molecules such as APX005M, ADC-1013 or the IgG2 mAb CP-870,893 do not include FcR engagement as MOA and are being examined in solid tumors alone or in combination with immune checkpoint inhibitors (NCT03123783; NCT02482168; NCT02379741). Positive results may lead to the investigation to hematological malignancies. In both types, it is expected that antitumor efficacy highly depends on the CD40 status of the tumor infiltrate, mainly tumor-specific CTLs and possibly TAMs. Accordingly, the direct tumoricidal effects depend highly on the CD40 expression of the tumor.

### Killer Cell Immunoglobulin-Like Receptor 3DL2 (KIR3DL2; CD158k)

This transmembrane glycoprotein belongs to the family of cell inhibitory receptors expressed by NK cells and subsets of CD8^+^ T-cells but not by most normal CD4^+^ T-cells. In contrast, KIR3DL2 expression is found in several CD4^+^ T malignancies, including SS, mycosis fungoides (MF) and anaplastic large cell lymphoma (ALCL) (98, 374). This receptor plays a dual role in the pathogenesis of these cancers: it acts as an inhibitory coreceptor which down-modulates CD3-dependent signaling events, and prevents programmed cell death. IPH-4102 is a humanized IgG1 antibody against KIR3DL2 whose potent antitumor properties *ex vivo* against SS and CTCL primary cells, and *in vivo* against KIR3DL2-positive tumor cells are achieved mainly through ADCC and ADCP. Only a minor contribution was attributed to the neutralization of the inhibitory receptor (98, 374). IPH-4102 advanced to phase I in 2015 (NCT02593045).

### CD70

This member of the TNF family is a receptor transiently expressed on activated T- and B-cells and on mature DC (375). Its ligand is CD27, another costimulatory receptor found on the surface of T-cells, B-cell, and NKs (376). The interaction of both molecules accelerates NK-mediated tumor clearance while generating an adaptive immune response (377). For this reason these NSLAs are being investigated in oncoimmunology. Whereas anti-CD27 antibodies, such as Varilumab (CDX-1127), just boost innate and adaptive antitumor responses, anti-CD70 antibodies also target tumor cells inducing direct cell killing. CD70 is expressed in several hematological malignancies that activate NF-κB pathways leading to proliferation and survival of malignant cells (378–380). In addition, CD70 seems to be involved in the recruitment of CD27-positive Tregs to the TME thus allowing tumor evasion (381). Despite most anti-CD70 mAbs under development are immunoconjugates (Table 6), one classical antibody is under clinical evaluation. ARGX-110 is a defucosylated IgG1 mAb with several different MOA (107). By neutralizing CD70–CD27 interactions it deprives cell growth signaling in tumor cells while inhibiting the activation and proliferation of CD27-positive Tregs. In addition, ARGX-110 displays enhanced ADCC and ADCP while preserving a strong CDC (107). The first phase I study (NCT02759250) in patients with advanced solid tumors expressing CD70 provided evidence of good tolerability of ARGX-110 and antitumor activity at all dose levels (108). Currently, a phase I–II study is recruiting patients to evaluate ARGX-110 efficacy in AML (NCT03030612).

### CD47 (Integrin-Associated Protein)

Phagocytosis is a complex process needed for programmed removal of apoptotic as well as IgG- or complement-opsonized cells that can be inhibited by the binding of the ubiquitous negative regulatory Ig receptor CD47 to the signal regulatory protein alpha (SIRPα), expressed on phagocytes and DCs (382, 383). CD47 was found to be universally expressed in human cancers where helps to prevent phagocytic elimination of tumor cells (118, 119). Notably, CD47 expression is preferentially found in AML-LSCs (384, 385) and negatively correlates with clinical outcome in AML, ALL, NHL, and MM (118, 119). The hypothesis that blocking CD47-SIRPα interactions would restore phagocytosis of tumor cells has been widely validated in primary human xenograft models treated with commercial and clinically developed anti-CD47 antibodies (118, 119, 384, 386–389). Based on this background, two novel anti-CD47 antibodies, Hu5F9-G4 and CC-900002, are being examined in several clinical studies (NCT02678338, NCT02953509, NCT02641002, NCT01410981, NCT02367196, NCT02663518), and many others have initiated clinical development, such as C47B222-(CHO).

CD47 is the first targeted receptor involved in phagocytosis, however, whether anti-CD47 MOA relies only in activation of immune cells or, in addition to immune cell activation, it strongly depends on Fc-mediated effector activities is a controversial issue. Based on preclinical investigations, it is assumed that these novel anti-CD47 mAbs impede CD47-SIRPα interactions leading to macrophage-mediated phagocytosis of B-NHL and AML cells, including LSC cells (118, 119). However, recent evidence suggests that the Fc region of the murine IgG1 (B6H12.2) and human IgG4 (Hu5F9-G4) are able to bind human and murine FcRs and mediate effector functions (118, 390, 391); hence, whether the therapeutic effect observed is due to solely blocking CD47 or to an opsonizing effect combined with CD47 blocking activity remains unclear. In this sense, antileukemic activity of the IgG1 antibody C47B222-(CHO) does not rely on CD47 neutralization but, on the contrary, depends on robust Fc effector functions such as ADCP and ADCC (118). Importantly, fusion proteins containing the high-affinity human SIPRα (known as CV1) only have antitumor activity when fused to IgG4, but not as SIRPα monomer (392, 393). Similarly, a SIRPα-Fc molecule known as TTI-621 demonstrated potent antileukemic activity as IgG1 Fc conjugate, but not with a Fc mutated to avoid effector functions (394). Furthermore, mice harboring inactivating mutations at the SIRPα cytoplasmic tail show similar growth and metastasis of implanted syngeneic melanoma tumor cells as wild-type mice, suggesting again that disruption of CD47-SIRPα alone does not yield an effective antitumor response (393).

Finally, another concern related to antibody-based CD47 therapy is tolerability. Except for C47B222-(CHO), anti-CD47 antibodies have been reported to cause platelet aggregation and red blood cell hemaglutination (118, 119). Therefore, it is not clear whether an optimal dosing strategy could be achieved that provides a therapeutic window with limited toxicity. Results from ongoing clinical studies will shed light on this issue.

## Antibody-Drug and Antibody-Radionuclide Conjugates (ADCs AND ARCs)

ADCs, along with ARCs, comprise the largest group of the non-canonical antibody formats in clinical studies for hematological malignancies. The principles of ADC/ARC activity and the considerations for their development, including the choice of antibody, drug and radionuclide, are beyond the scope of this review and have been discussed elsewhere (3, 395, 396). However, as the most advanced ADCs in the clinic are directed to hematological indications, a brief account of this landscape with some examples of ADCs which target LSA and NLSAs are given in Tables 5 and 6, including the first ADCs developed and approved by the FDA: gemtuzumab ozogamicin, now discontinued, and brentuximab vedotin.

**Table 5:** Characteristics of ADCs and ARCs directed to LSAs.

Target	mAb (commercial name/originator)	IgG class	Conjugate	Active indications in HMs (highest phase)	Reference
CD33	Gemtuzumab ozogamicin (*Mylotarg1*/UCB)	Hz IgG4	Calicheamicin	Approved (AML, W)	(120, 121)
				IV (AML/NCT00161668 ^a^C)	
				III (AML/NCT00136084 ^b^C, NCT00049517 ^b^ANR, NCT00372593 ^b^C)	
				II (CML/NCT01019317 ^b^C)	
				II (APL/NCT00413166 ^b^C)	
				I–II (CLL/NCT00038831 ^b^C)	

	Vadastuximab talirine, SGN-CD33A (Seattle Genetics)	Hz IgG1	PBD	III (AML/NCT02785900 ^b^T)	(122)
				I–II (MDS/NCT02706899 ^b^S)	
				I (APL/NCT01902329 ^b^ANR)	

	IMGN-779 (ImmunoGen)	N/A	DGN462	I (AML/NCT02674763 ^a^R)	(123)

	AVE9633 (ImmunoGen)	Hz IgG1	DM4	I (AML/NCT00543972 ^a^T) disc.	(124)

	^225^Ac-HuM195, Lintuzumab-Ac225 (PDL BioPharma)	Hz IgG1	Actinium-225	III (AML/NCT00006045 ^b^U)	(125)
				II (CML/NCT00002800 ^b^C)	
				II (APL/NCT00002609 ^b^C)	
				II (MDS/NCT00997243 ^b^T)	
				I (MM/NCT02998047 ^a^R)	

CD19	Coltuximab ravtansine, SAR3419 (Sanofi, ImmunoGen)	Hz IgG1	DM4	II (DLBCL/NCT01470456 ^b^C, NCT01472887 ^a^C)	(126)
				II (B-ALL/NCT01440179 ^a^T) disc.	
				I (B-NHL/NCT00796731 ^a^C)	

	Denintuzumab mafodotin, SGN-CD19A (Seattle Genetics)	Hz IgG1	MMAF	II (DLBCL/FL/NCT02855359 ^b^R)	(127)
				I (B-ALL/NCT01786096 ^b^C)	

CD20	131-I-tositumomab (*Bexxar/*Corixa Corp.)	m IgG1	Iodine 131	Approved (B-NHL, W)	(128)
				II (MCL/NCT00022945^b^ C) disc.	
				III (FL/NCT00268983^b^ C)	
				II (CLL/NCT00476047^b^ C)	

	Y90-Ibritumomab tiuxetan (*Zevalin/*Biogen Idec)	m IgG1	Yttrium 90	Approved/B-NHL	(129)
				III (FL/NCT02063685^b^ R)	
				III (MCL/NCT01510184^a^ T) disc.	
				III (DLBCL/NCT02366663^b^ T) disc.	
				II (CLL/NCT00119392^a^ ANR)	
				II (MM/NCT01207765^a^ T) disc.	

CD22	Inotuzumab ozogamicin, CMC-544 (*Besponsa*/Celltech Group)	Hz IgG4	Calicheamicin	Approved/ALL	(130)
				III (AML/NCT03150693^b^ R)	
				III (DLBCL/NCT01232556^b^ T) disc.	
				III (FL/NCT00562965^b^ T)	

	Pinatuzumab vedotin, RG7593 (Genentech)	Hz IgG1	MMAE	I–II (B-NHL/NCT01691898^b^ ANR) disc.	(131)

	Epratuzumab-SN38 (Immunomedics)	Hz IgG1	SN-38	Preclinical (B-NHL, B-ALL)	(132)

	Epratuzumab I-131 (Immunomedics)	Hz IgG1	Iodine 131	Preclinical (B-NHL) disc.	(133)

	Moxetumomab pasudotox, CAT-3888 (NCI)	Fv	PE-38	III (HCL/NCT01829711^a^ ANR)	(134)
				II (B-ALL/NCT02338050^a^ T)	
				I/II (CLL/B-NHL/NCT01030536^a^ C) disc.	

CD79b	Polatuzumab vedotin, RG-759 (Genentech)	Hz IgG1	MMAE	II (DLBCL/NCT01992653^b^ ANR)	(135)
				I/II (FL/NCT01691898^b^ ANR)	
				I (CLL/NCT01290549^b^ C)	

CD138	Indatuximab ravtansine, BT-062 (BioTest AG)	Ch IgG4	DM4	II (MM/NCT01638936^a^ ANR NCT01001442^a^ C)	(136)

*Antibodies that reached clinical studies*.

*^a^Monotherapy*.

*^b^Combined therapy*.

*ADCs, antibody-drug conjugates; ARC, antibody-radionuclide conjugates; mAb, monoclonal antibody; HMs, hematological malignancies; Ch, human–mouse chimeric; Fh, fully human; Hz, humanized; m, mouse; Glyco-Fc, glycoengineered Fc fragment; N/A, not available; Fv, fragment variable; AML, acute myeloid leukemia; APL, acute prolymphocytic leukemia; CML, chronic myeloid leukemia; CLL, chronic lymphocytic leukemia; MDS, myelodysplastic syndrome; MM, multiple myeloma; DLBCL, diffuse large B-cell lymphoma; B-ALL, B-cell acute lymphoblastic leukemia; B-NHL, B-cell non-Hodgkin’s lymphoma; FL, follicular lymphoma; MCL, mantle cell lymphoma; HCL, hairy cell leukemia; disc., discontinued in hematological malignancies; NCT, number of clinical trial (clinicaltrials.gov); C, completed; R, recruiting; T, terminated; ANR, active non-recruiting; NYR, not yet recruiting; T, terminated; S, suspended; W, withdrawn; U, unknown; PBD, pyrrolobenzodiazepine dimmers; MMA, monomethyl auristatin; PE-38 *Pseudomonas aeruginosa* exotoxin A; DGN462, a DNA-alkylating payload; DM4, a cytotoxic maytansinoid; SN-38, an active metabolite of irinotecan*.

**Table 6:** Characteristics of ADCs and ARCs directed to NLSAs.

Target	mAb (commercial name/originator)	IgG class	Conjugate	Active indications in HMs (highest phase)	Reference
CD25	ADCT-301, HuMax-TAC-PBD (ADC Therapeutics; Genmab)	Fh IgG1	PBD	I (AML/B-ALL/NCT02588092 ^a^R)	(137)
				I (HL, B-NHL, PTCL, CTCL/NCT02432235 ^a^R)	

	IMTOX-25, RFT5-DGA (XOMA)	mIgG1	DGA	II (ATL/NCT01378871^a^ C) disc.	(138)
				II (T-NHL/NCT00667017^a^ C) disc.	

	anti-Tac(Fv)-PE38, LMB-2 (NCI)	Fv	PE-38	II (HCL/NCT00321555 ^a^ANR, NCT00923013 ^b^R)	(139)
				II (CTCL/NCT00080535 ^a^C)	
				II (CLL/NCT00077922 ^a^C) disc.	
				I–II (ATLL/NCT00924170 ^b^ANR)	

CD30	Brentuximab vedotin, SGN-35 (*Adcetris*/Seattle Genetics)	Ch IgG1	MMAE	Approved (ALCL, HL)	(140)
				Preregistration (CTCL)	
				III (CTCL/NCT01578499 ^a^ANR)	
				III (SS/NCT01578499 ^a^ANR)	
				III (T-cell lymphoma/NCT01777152 ^a^ANR)	
				II (DLBCL/NCT02734771 ^b^R, NCT01925612 ^b^T)	
				II (AML/NCT01461538 ^b^C)	
				II (FL, MZL/NCT02623920 ^b^W, NCT02594163 ^b^ANR)	
				II (MF/SS/PTCL/NCT01352520 ^a^ANR, NCT03113500 ^b^R)	
				I–II (NK-T lymphoma/NCT03246750 ^b^NYR)	

CD37	AGS67E (Agensys)	Fh IgG2	MMAE	I (AML/NCT02610062 ^a^R)	(79)
				I (B-NHL/NCT02175433 ^a^R)	

	IMGN529, K7153A (ImmunoGen)	Hz IgG1	DM1	II (DLBCL/NCT02564744 ^b^R)	(141)
				I (CLL, B-NHL/NCT01534715 ^a^C)	

	177Lu-tetulomab, lilotomab (*Betalutin*/Nordic Nanovector)	Mouse N/A	Lutetium 177	I–II (B-NHL/NCT01796171 ^a^R)	(142)
				I (DLBCL/NCT02658968 ^a^R)	

CD56	Lorvotuzumab mertansine, IMGN-901 (ImmunoGen)	Hz IgG1	DM1	II (AML, NK-cell leukemia, B-ALL CML, MF/NCT02420873^a^ C)	(143)
				I (MM/NCT00991562 ^b^C/NCT00346255 ^a^C)	

CD70	Vorsetuzumab mafodotin, SGN-75 (Seattle Genetics)	Hz IgG1	MMAF	I (B-NHL/NCT01015911^a^ C) disc.	(144)
				Lack of efficacy; second generation SGN-70A	

	SGN-70A (Seattle Genetics)	Hz IgG1	PBD	I (DLBCL, MCL, FL/NCT02216890^a^ C) disc. (superseding SGN-75)	(145)

	MDX-1203 (Medarex)	Fh IgG1	MED-2460	I (B-NHL/NCT00944905^a^ C)	(146)

CD74	Milatuzumab-doxorubicin, IMMU-110 (Immunomedics)	Hz IgG1	Doxorubicin	I–II (MM/NCT01101594^a^ C)	(147)

CD117	LOP-628 (Novartis)	Hz IgG1	Maytansine	I (AML/NCT02221505^a^ T) disc.	

*Antibodies that reached clinical studies*.

*^a^Monotherapy*.

*^b^Combined therapy*.

*ADCs, antibody-drug conjugates; ARC, antibody-radionuclide conjugates; mAb, monoclonal antibody; HMs, hematological malignancies; Ch, human–mouse chimeric; Fh, fully human; Hz, humanized; m, mouse; N/A, not available; Fv, fragment variable; AML, acute myeloid leukemia; HL, Hodgkin’s lymphoma; B-ALL, B-cell acute lymphoblastic leukemia; B-NHL, B-cell non-Hodgkin’s lymphoma; PTCL, peripheral T-cell lymphoma; CTCL, cutaneous T-cell lymphoma; ATL, adult T-cell leukemia and lymphoma; HCL, hairy cell leukemia; CLL, chronic lymphocytic leukemia; ALCL, anaplastic large-cell lymphoma; SS, Sézary syndrome; DLBCL, diffuse large B-cell lymphoma; FL, follicular lymphoma; MZL, marginal zone lymphoma; MF, mycosis fungoides; CML, chronic myeloid leukemia; MM, multiple myeloma; MCL, mantle cell lymphoma; disc., discontinued in hematological malignancies; NCT, number of clinical trial (clinicaltrials.gov); C, completed; R, recruiting; T, terminated; ANR, active non-recruiting; NYR, not yet recruiting; T, terminated; S, suspended; W, withdrawn; U, unknown; PBD, pyrrolobenzodiazepine dimmers; DGA (deglyscosylated ricin A-chain); PE-38 *Pseudomonas aeruginosa* exotoxin A; MMA, monomethyl auristatin; DM1, a cytotoxic maytansinoid; MED-2460, a DNA-alkylating payload*.

## Conclusion

From rituximab, the first mAb approved in 1997 to treat cancer, to the recently approved daratumumab and elotuzumab to treat MM, several antibodies have changed the clinical practice and have transformed the therapeutic landscape of hematological malignancies. However, as we have shown, this number is extremely low compared with the total number of antibodies being studied in clinical trials. Today, most of the antibodies already approved target LSAs, whereas the majority of agents in development for hematological malignancies targets NLSAs. This situation reflects a paradigm shift in the criteria followed to select a target to develop as novel immune therapy in blood malignancies care. The impact of this change is still unknown in disorders where mAbs targeting LSAs were not used, or resulted to be ineffective, such as MM, AML, or T-NHL. In this respect, novel antibodies directly targeting NLSAs in MM or AML are a profound change compared with earlier treatment approaches. Remarkably, today most of the therapeutic antibodies under development target both disorders. Nonetheless, in other diseases, such as T-cell or NK-cells malignancies, the scenario is not so promising since few candidates are in clinical development, and many of them show lack of activity in these entities. For example, while patients with B-NHL may benefit from immune checkpoint drugs, it is likely that patients with T-lymphomas will not. However, efforts must be maintained. NLSAs constitute a bigger group of molecules than LSAs, and for this reason the number of antibodies showing successful results is likely to be higher.

Most of the antibodies reviewed in this work, used as single agent or in combined regimens, are not better than standard treatments. In some cases the selected NLSA is not appropriate. For instance, targeting soluble factors is a successful approach in immunological disorders that seems to be ineffective against hematological malignancies, likely due to the disseminated nature of these cancers. In other cases, the antibody under evaluation may not display all its potential or may not trigger the right MOA. In this respect, directed modifications both in the Fv and Fc may reverse the lack of activity. Moreover, combining antibodies with other drugs, without previous strong supporting evidence, may negatively affect the outcome in clinical settings. Unfavorable combination regimens may impair the main MOA of a single antibody, or even highly pretreated patients may harbor the unfavorable background themselves. To evaluate a novel antibody, the selection of patients must take into account several factors including sensitivity and tolerability to prior treatments, disease stage and cytogenetic profile. Finally, one of the most important issues in the evaluation of antibodies targeting NLSAs expressed in different disorders is the selection of the optimal dose, which is not usually based on biological criteria. As we have learnt from some antibodies targeting LSAs, benefits within a wide range of identical lineage disorders cannot be obtained using the same dosing schedule. The uncertainty over the optimal dosing schedule is even higher when a particular mAb is examined in diseases differing not only in maturation stages but most importantly in different lineages or even, in different tissues.

Far beyond these explanations, the abundance of novel targeted agents draw a promising and consolidated landscape in hematological malignancies. Hopefully, in the near future, clinicians may consider different standard treatments for a given disease as different treatments may be available and may be tailored for molecularly defined responsive groups. In the coming years, unmet medical needs in a wide variety of conditions should be reduced and patient choices for antibody therapeutics should increase in short- as well as long-term. The optimal integration of mAbs and other novel agents with current treatment strategies will require intelligent, rather than commercially driven, preclinical, and clinical design over the coming years.

## Author Contributions

CC-M reviewed the literature, prepared the tables, and wrote the manuscript. AA-S and BS-C reviewed the literature and drafted the work. CM-C reviewed the literature and wrote the manuscript.

## Conflict of Interest Statement

CM-C received research funding from BMS and IMMED.S.L. and has filed patents on targeting of the CCR7 chemokine receptor as cancer therapy. CC-M is an employee of IMMED. The remaining authors declare no conflict of interest.
